# Berberine: Botanical Occurrence, Traditional Uses, Extraction Methods, and Relevance in Cardiovascular, Metabolic, Hepatic, and Renal Disorders

**DOI:** 10.3389/fphar.2018.00557

**Published:** 2018-08-21

**Authors:** Maria A. Neag, Andrei Mocan, Javier Echeverría, Raluca M. Pop, Corina I. Bocsan, Gianina Crişan, Anca D. Buzoianu

**Affiliations:** ^1^Department of Pharmacology, Toxicology and Clinical Pharmacology, “Iuliu Hatieganu” University of Medicine and Pharmacy, Cluj-Napoca, Romania; ^2^Department of Pharmaceutical Botany, “Iuliu Hatieganu” University of Medicine and Pharmacy, Cluj-Napoca, Romania; ^3^Department of Environmental Sciences, Universidad de Santiago de Chile, Santiago de Chile, Chile

**Keywords:** berberine, botanical occurrence, traditional uses, extraction methods, biological activities

## Abstract

Berberine-containing plants have been traditionally used in different parts of the world for the treatment of inflammatory disorders, skin diseases, wound healing, reducing fevers, affections of eyes, treatment of tumors, digestive and respiratory diseases, and microbial pathologies. The physico-chemical properties of berberine contribute to the high diversity of extraction and detection methods. Considering its particularities this review describes various methods mentioned in the literature so far with reference to the most important factors influencing berberine extraction. Further, the common separation and detection methods like thin layer chromatography, high performance liquid chromatography, and mass spectrometry are discussed in order to give a complex overview of the existing methods. Additionally, many clinical and experimental studies suggest that berberine has several pharmacological properties, such as immunomodulatory, antioxidative, cardioprotective, hepatoprotective, and renoprotective effects. This review summarizes the main information about botanical occurrence, traditional uses, extraction methods, and pharmacological effects of berberine and berberine-containing plants.

## Introduction

### Berberine

**Figure 1:**
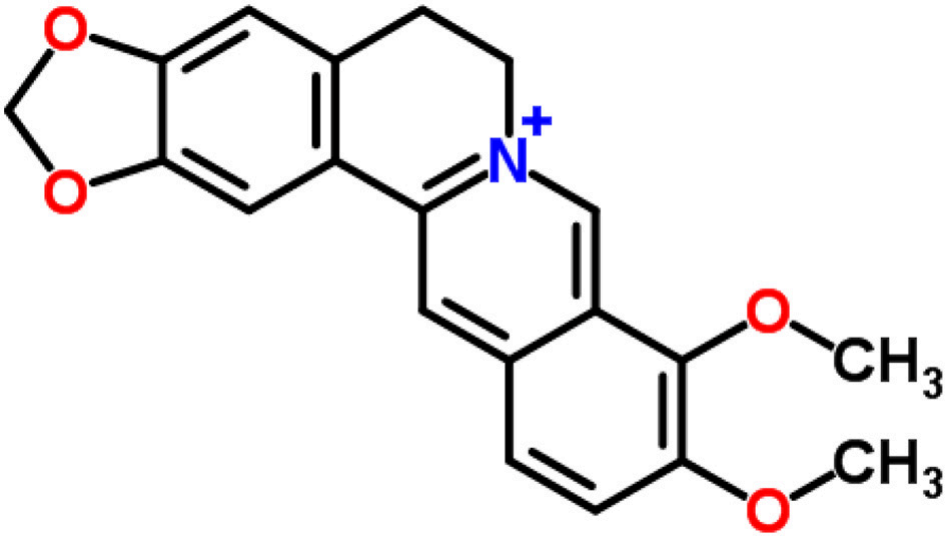
Berberine structure (according to ChemSpider database).

Berberine(5,6-dihydro-9,10-dimethoxybenzo[g]-1,3-benzodioxolo[5,6-a] quinolizinium) Figure 1, is a nonbasic and quaternary benzylisoquinoline alkaloid, a relevant molecule in pharmacology and medicinal chemistry. Indeed, it is known as a very important natural alkaloid for the synthesis of several bioactive derivatives by means of condensation, modification, and substitution of functional groups in strategic positions for the design of new, selective, and powerful drugs (Chen et al., 2005).

## Traditional use of berberine-containing species

In the Berberidaceae family, the genus *Berberis* comprises of ~450–500 species, which represent the main natural source of berberine. Plants of this genus are used against inflammation, infectious diseases, diabetes, constipation, and other pathologies (Singh A. et al., 2010). The oldest evidence of using barberry fruit (*Berberis vulgaris*) as a blood purifying agent was written on the clay tablets in the library of Assyrian emperor Asurbanipal during 650 BC (Karimov, 1993). In Asia, the extensive use of the stem, stem bark, roots, and root bark of plants rich in berberine, particularly *Berberis* species, has more than 3000 years of history. Moreover, they have been used as raw material or as an important ingredient in Ayurvedic and traditional Chinese medicine (Birdsall, 1997; Kirtikar and Basu, 1998; Gupta and Tandon, 2004; Kulkarni and Dhir, 2010). In Ayurveda, *Berberis* species have been traditionally used for the treatment of a wide range of infections of the ear, eye, and mouth, for quick healing of wounds, curing hemorrhoids, indigestion and dysentery, or treatment of uterine and vaginal disorders. It has also been used to reduce obesity, and as an antidote for the treatment of scorpion sting or snakebite (Dev, 2006). Berberine extracts and decoctions are traditionally used for their activities against a variety of microorganisms including bacteria, viruses, fungi, protozoa, helminthes, in Ayurvedic, Chinese, and Middle-Eastern folk medicines (Tang et al., 2009; Gu et al., 2010).

In Yunani medicine, *Berberis asiatica* has multiple uses, such as for the treatment of asthma, eye sores, jaundice, skin pigmentation, and toothache, as well as for favoring the elimination of inflammation and swelling, and for drying ulcers (Kirtikar and Basu, 1998). Decoction of the roots, and stem barks originating from *Berberis aristata, B. chitria*, and *B. lycium* (Indian *Berberis* species), have been used as domestic treatment of conjunctivitis or other ophthalmic diseases, enlarged liver and spleen, hemorrhages, jaundice, and skin diseases like ulcers (Rajasekaran and Kumar, 2009). On the other hand, the use of decoction of Indian barberry mixed with honey has also been reported for the treatment of jaundice. Additionally, it has been reported the use of decoction of Indian barberry and *Emblic myrobalan* mixed with honey in the cure of urinary disorders as painful micturition (Kirtikar and Basu, 1998). Numerous studies dealing with its antimicrobial and antiprotozoal activities against different types of infectious organisms (Vennerstrom et al., 1990; Stermitz et al., 2000; Bahar et al., 2011) have been assessed so far. Moreover, it has been used to treat diarrhea (Chen et al., 2014) and intestinal parasites since ancient times in China (Singh and Mahajan, 2013), and the Eastern hemisphere, while in China it is also used for treating diabetes (Li et al., 2004).

Nowadays, a significant number of dietary supplements based on plants containing berberine (Kataoka et al., 2008) are used for reducing fever, common cold, respiratory infections, and influenza (Fabricant and Farnsworth, 2001). Another reported use for berberine-containing plants is their application as an astringent agent to lower the tone of the skin. Also, positive effects were observed on the mucous membranes of the upper respiratory tract and gastrointestinal system with effects on the associated ailments (Chen et al., 2014; Yu et al., 2016).

In southern South America leaves and bark of species of the genus *Berberis* are used in traditional medicine administered for mountain sickness, infections, and fever (San Martín, 1983; Houghton and Manby, 1985; Anesini and Perez, 1993).

Furthermore, there are other genera which contain berberine. The genus *Mahonia* comprises of several species that contain berberine. Within them, *M. aquifolium* has been traditionally used for various skin conditions. Due to its main alkaloid (berberine), is known to be used in Asian medicine for its antimicrobial activity. *Coptidis rhizoma* (rhizomes of *Coptis chinensis*), another plant which contains berberine, is a famous herb very frequently used in traditional Chinese medicine for the elimination of toxins, “damp-heat syndromes”, “purge fire”, and to “clear heat in the liver” (Tang et al., 2009). Table 1 gathers a synthesis of the main traditional uses of species containing berberine.

**Table 1:** Traditional uses of berberine-containing species.

**Family**	**Scientific name**	**Traditional uses**	**References**
Annonaceae	*Annickia chlorantha* (Oliv.) Setten & Maas (ex-*Enantia chlorantha* Oliv.)	Treat jaundice, hepatitis A, B, C, and D, conjunctivitis, leishmaniasis, medicine for cuts and infected wounds, sores and ulcers, antipyretic for various fevers, tuberculosis, vomiting of blood, urinary tract infections, treatment of fatigue, rheumatism, treat malaria symptoms, aches, wounds, boils, vomiting, yellow bitter, chills, sore, spleen in children and body pains, skin ailments, intercostal pain and to promote conception, intestinal worms, intestinal spasms, malaria and sexual asthenia, treat coughs and wounds; rickettsia fever, treat of sleeping sickness and dysentery, hemostatic and rickettsia, treat yellow fever and typhoid fever, treat diabetes, treat syphilis, and other infectious diseases, poliomyelitis, treat hypertension, treat HIV and prostate cancer	Oliver, 1960; Sandberg, 1965; Bouquet, 1969; Hamonniere et al., 1975; Onwuanibe, 1979; Burkill, 1985; Gill and Akinwumi, 1986; Gbile et al., 1988; Vennerstrom and Klayman, 1988; Vennerstrom et al., 1990; Adjanohoun et al., 1996; Nguimatsia et al., 1998; Kayode, 2006; Odugbemi et al., 2007; Ehiagbonare and Onyibe, 2008; Jiofack et al., 2008, 2009; Kadiri, 2008; Ogbonna et al., 2008; Olowokudejo et al., 2008; Betti and Lejoly, 2009; Ndenecho, 2009; Adeyemi et al., 2010; Noumi, 2010; Noumi and Anguessin, 2010; Noumi and Yumdinguetmun, 2010; Bele et al., 2011; Din et al., 2011; Ngono Ngane et al., 2011; Oladunmoye and Kehinde, 2011; Gbolade, 2012; Musuyu Muganza et al., 2012; Tsabang et al., 2012; Betti et al., 2013; Borokini et al., 2013; Fongod, 2014; Ishola et al., 2014; Ohemu et al., 2014
	*Annickia pilosa* (Exell) Setten & Maas (ex-*Enantia pilosa* Exell)	Medicine for cuts	Versteegh and Sosef, 2007
	*Annickia polycarpa* (DC.) Setten & Maas ex I.M.Turner (ex-*Enantia polycarpa* (DC.) Engl. & Diels)	Treat cuts, antiseptic to treat sores, stomach ulcers, leprosy and ophthalmia, treatment of skin infections and sores, treat jaundice, and treat fever including malaria and to promote wound healing, against intestinal problems	Irvine, 1961; Bouquet and Debray, 1974; Ajali, 2000; Govindasamy et al., 2007; Versteegh and Sosef, 2007
	*Rollinia mucosa* (Jacq.) Baill.	Treat of tumors	Hartwell, 1982
	*Xylopia polycarpa* (DC.) Oliv.	Treat wounds, ulcers, leprosy, rheumatism, stomach and gall-bladder problems, eye diseases, for conception, diarrhea, malaria, fevers and sleeping disorders	Neuwinger, 1996
Berberidaceae	*Berberis actinacantha* Mart.	Antipyretic	San Martín, 1983
	*Berberis aquifolium* Pursh	Skin conditions, treat eczema, acne, conjunctivitis and herpes, alleviate the symptoms of psoriasis, treat diarrhea and in higher doses to treat constipation, improvement of blood flow to the liver, stimulate intestinal secretions and bile flow, treat jaundice, hepatitis, cirrhosis and general digestive problems, treatment of gall bladder disease, hemorrhages and a few forms of cancer, fungal infections, dysentery, anti-inflammatory properties, stomach problems, sore womb following childbirth and/or menstruation	King, 1898; Ritch-Krc et al., 1996
	*Berberis aristata* DC.	Treat allergies, metabolic disorders, ophthalmia, and other eye diseases, treat bleeding piles, anti-osteoporosis, treat skin diseases, menorrhagia, fever, diarrhea, dysentery, cholera, jaundice, ear and urinary tract infections, anti-bacterial, anti-fungal, anti-inflammatory, analgesic, anti-pyretic, jaundice, piles, malaria, laxative, anti-scorbutic, anti-diabetic, and anti-hepatopathic	Bhattacharjee et al., 1980; Duke and Beckstrom-Sternberg, 1994; Küpeli et al., 2002; Acharya and Rokaya, 2005; Chhetri et al., 2005; Kunwar and Adhikari, 2005; Sharma et al., 2005; Joshi and Joshi, 2007; Meena et al., 2009; Shahid et al., 2009; Phondani et al., 2010; Saraf et al., 2010; Tiwary et al., 2010; Sati and Joshi, 2011; Yogesh et al., 2011
	*Berberis asiatica* Roxb. ex DC.	Jaundice, diabetes mellitus, wound healing, asthma; drying unhealthy ulcers, anti-inflammatory, swelling, treat pneumococcal infections, eye (conjunctivitis) and ear diseases, rheumatism, fever, stomach disorders, skin disease (hyperpigmentation), malarial fever, laxative, teeth problems (toothache), and headache	Watt, 1883; Kirtikar and Basu, 1933; Samhita, 1963; Hashmi and Hafiz, 1986; Bhandari et al., 2000; Shah and Khan, 2006; Uniyal et al., 2006; Uprety et al., 2010; Maithani et al., 2014
	*Berberis buxifolia* Lam.	Treat infections	Anesini and Perez, 1993; Mølgaard et al., 2011
	*Berberis chitria* Buch.-Ham. ex Lindl.	Treat skin disease, jaundice, rheumatism, affection of eyes (household treatment for conjunctivitis, ophthalmic, bleeding piles), ulcers, skin diseases, enlarged liver and spleen	Watt, 1883; Kirtikar and Basu, 1933; Sir and Chopra, 1958
	*Berberis darwinii* Hook.	Antipyrectic, anti-inflammatory, treat stomach pains, indigestion, and colitis	Montes and Wilkomirsky, 1987
	*Berberis empetrifolia* Lam.	Treat mountain sickness	San Martín, 1983
	*Berberis integerrima* Bunge.	Antipyretic, treat diabetes, bone fractures, rheumatism, radiculitis, heart pain, stomach aches, kidney stones, tuberculosis, chest pain, headaches, constipation, and wound	Khalmatov, 1964; Khodzhimatov, 1989; Baharvand-Ahmadi et al., 2016
	*Berberis jaeschkeana* C. K. Schneid.	Treat eye diseases	Kala, 2006
	*Berberis koreana* Palib.	Antipyretic, treat gastroenteritis, sore throats, and conjunctivitis	Ahn, 2003
	*Berberis leschenaultia* Wall. ex Wight & Arn.	Antipyretic, cold and complications during post-natal period	Rajan and Sethuraman, 1992
	*Berberis libanotica* Ehrenb. ex C. K. Schneid.	Treat rheumatic and neuralgic diseases, anti-inflammatory, treat arthritis and muscular pain	El Beyrouthy et al., 2008; Esseily et al., 2012
	*Berberis lycium* Royle	Treat eye diseases, febrifuge, jaundice, diarrhoea, menorrhagia, piles, backache, dysentery, earache, fracture, eye ache, pimples, boils, wound healing, cough and throat pain, intestinal colic, diabetes, throat pain, scabies, bone fractures, sun blindness, against stomachache and intestinal problems	Zaman and Khan, 1970; ul Haq and Hussain, 1993; Bushra et al., 2000; Kaur and Miani, 2001; Hamayun et al., 2003; Ahmed et al., 2004; Abbasi et al., 2005, 2009, 2010; Shah and Khan, 2006; Zabihullah et al., 2006; Hussain et al., 2008; Sood et al., 2010
	*Berberis microphylla* G. Forst. (ex-*Berberis heterophylla* Juss. ex Poir.)	Febrifuge, anti-inflammatory and treat diarrhea	Muñoz, 2001
	*Berberis oblonga* (Regel) C. K. Schneid	Heart tonic, treat neurasthenia, antipyretic, antidiarrheal, treat rheumatism, eye diseases and wounds of the mouth, jaundice, stomach aches, back pain and arthralgia	Khalmatov, 1964; Sezik et al., 2004; Pak, 2005
	*Berberis petiolaris* Wall. ex G. Don	Treat malarial fever, diarrhea, conjunctivitis, and jaundice	Karimov, 1993
	*Berberis pseudumbellata* R. Parker	Diuretic, treat jaundice, intestinal disorders, eye diseases, oxytocic and throat ache, stomach problems and ulcers	Kala, 2006; Khan and Khatoon, 2007; Singh et al., 2009; Khan et al., 2016
	*Berberis thunbergii* DC.	Anti-inflammatory	Küpeli et al., 2002
	*Berberis tinctoria* Lesch.	Antimicrobial for skin disease, jaundice, affection of eyes, treat menorrhagia, diarrhea, and rheumatism	Fyson, 1975; Satyavati et al., 1987
	*Berberis umbellata* Wall. ex G. Don	Treating fever, jaundice, nausea, eye disorders and skin problems, tonic	Singh et al., 2012
	*Berberis vulgaris* L.	Antiarrhythmic, sedative, anticancer, heal internal injuries, remove kidney stones, treat sore throat and fever	Tantaquidgeon, 1928; Chaudhury et al., 1980; Zovko Koncić et al., 2010
	*Caulophyllum thalictroides* (L.) Michaux	Menstrual cramps, relieve the pain of childbirth, promote prompt delivery, treat colics, cramps, hysteria, rheumatism, uterine stimulant, inducer of menstruation, and antispasmodic	Castleman, 1991; Hutchens, 1992
	*Jeffersonia diphylla* (L.) Pers.	Antispasmodic, diuretic, emetic, expectorant, treat diarrhea, dropsy, gravel and urinary problems, emetic, expectorant, treat sores, ulcers and inflamed parts	Uphof, 1959; Duke and Ayensu, 1985; Foster and Duke, 1990; Coffey, 1993; Moerman, 1998; Lust, 2014
	*Mahonia fortunei* (Lindl.) Fedde	Anticancer, febrifuge, antiodontalgic, treat testicular swelling and arthritic pain	Duke and Ayensu, 1985; He and Mu, 2015
	*Mahonia napaulensis* DC.	Diuretic, demulcent, treat dysentery and inflammations of the eyes	Chopra et al., 1986; Manandhar, 2002
	*Nandina domestica* Thunb.	Antitussive, astringent, febrifuge, stomachic and tonic, treat of fever in influenza, acute bronchitis, whooping cough, indigestion, acute gastro-enteritis, tooth abscess, pain in the bones, muscles and traumatic injuries, and antirheumatic	Kariyone and Koiso, 1971; Duke and Ayensu, 1985; Fogarty, 1990
	*Sinopodophyllum hexandrum* (Royle) T. S. Ying	Regulate menstruation, promote the circulation of blood, treat amenorrhea, difficult labor and retention of dead fetus or placenta	Kong et al., 2010
Menispermaceae	*Tinospora sinensis* (Lour.) Merr (ex-*Tinospora cordifolia* (Willd.) Miers)	Tonic, antiperiodic, anti-spasmodic, anti-inflammatory, antiarthritic, anti-allergic, anti-diabetic, improve the immune system, antistress, anti-leprotic and anti-malarial activities	Singh et al., 2003
Papaveraceae	*Argemone albiflora* Hornem (ex-*Argemone alba* F. Lestib.)	Anthydropic, cathartic, diaphoretic, diuretic, demulcent, emetic, purgative, treat jaundice, skin ailments, colds, colics and wounds	Smyth, 1903; Foster and Duke, 1990
	*Argemone mexicana* L.	Analgesic, antispasmodic, sedative, treat warts, cold sores, cutaneous affections, skin diseases, itches, treat cataracts, treat dropsy, jaundice, treat chronic skin diseases, expectorant, treat coughs and chest complaints, demulcent, emetic, expectorant, laxative and antidote to snake poisoning	Uphof, 1959; Pesman, 1962; Usher, 1974; Stuart and Smith, 1977; Emboden, 1979; Chopra et al., 1986; Coffey, 1993; Chevallier, 1996
	*Argemone platyceras* L.	Treat respiratory ailments as asthma, cough, bronchitis and pneumonia	Emes et al., 1994
	*Bocconia frutescens* L.	Treat skin conditions (ulcers and eruptions) and respiratory tract infections (bronchistis and tuberculosis)	Martinez, 1977, 1984
	*Chelidonium majus* L.	Treat ophthalmic diseases (remove films from the cornea of the eye), mild sedative, antispasmodic, relaxing the muscles of the bronchial tubes and intestines, treat warts, alterative, anodyne, antispasmodic, cholagogue, diaphoretic, diuretic, hydrogogue, narcotic, purgative, treat bronchitis, whooping cough, asthma, jaundice, gallstones and gallbladder pains, anticancer, analgesic, treat stomach ulcer, treat get rid of warts, ringworm and corns	Launert, 1981; Grieve, 1984; Phillips and Foy, 1990; Phillips and Rix, 1991; Chevallier, 1996; Lust, 2014
	*Corydalis solida* subsp. *brachylova*	Anodyne, antibacterial, antispasmodic, hallucinogenic, calm the nerves, sedative for insomnia, CNS stimulant, painkiller, treat painful menstruation, lowering the blood pressure, traumatic injury and lumbago	Launert, 1981; Bown, 1995
	*Corydalis solida* subsp. *slivenensis* (Velen.) Hayek (ex-*Corydalis slivenensis* Velen.)		
	*Corydalis solida* subsp. *tauri cola*		
	*Corydalis turtschaninovii* Besser (ex-*Corydalis ternata* (Nakai) Nakai)	Treat memory dysfunction, treat gastric, duodenal ulcer, cardiac arrhythmia disease, rheumatism and dysmenorrhea	Tang and Eisenbrand, 1992; Kamigauchi and Iwasa, 1994; Orhan et al., 2004; Houghton et al., 2006
	*Eschscholzia californica* Cham.	Sedative, diuretic, relieve pain, relax spasms, promote perspiration, treat nervous tension, anxiety, insomnia, urinary incontinence (especially in children), narcotic, relieve toothache, antispasmodic, analgesic and suppress the flow of milk in lactating women	Coffey, 1993; Bown, 1995; Chevallier, 1996; Moerman, 1998
	*Glaucium corniculatum* (L.) Rud. subsp. *corniculatum*	Reduce warts, antitusive, treat CNS disturbances, sedative, cooling, and mild laxative	Al-Douri, 2000; Al-Qura'n, 2009; Hayta et al., 2014
	*Macleaya cordata* (Willd.) R.Br.	Analgesic, antioedemic, carminative, depurative, diuretic, treat insect bites, and ringworm	Grieve, 1984; Duke and Ayensu, 1985
	*Macleaya microcarpa* (Maxim.) Fedde	Treat some skin diseases and inflammation	Deng and Qin, 2010
	*Papaver dubium* L.	Sudorific, diuretic, expectorant and ophthalmia	Chopra et al., 1986
	*Papaver dubium* var. *lecoquii*		
	*Papaver rhoeas* L. var. *chelidonioides*	Ailments in the elderly and children, mild pain reliever, treat irritable coughs, reduce nervous over-activity, anodyne, emollient, emmenagogue, expectorant, hypnotic, slightly narcotic, sedative, treat bronchial complaints and coughs, insomnia, poor digestion, nervous digestive disorders and minor painful conditions, treat jaundice, fevers, and anticancer	Uphof, 1959; Launert, 1981; Grieve, 1984; Duke and Ayensu, 1985; Phillips and Foy, 1990; Bown, 1995; Chevallier, 1996
	*Papaver hybridum* L.	Treat dermatologic diseases, anti-infective, diuretic, sedative, and antitussive	Rivera Núñez and Obon de Castro, 1996; Ali et al., 2018
Ranunculaceae	*Coptis chinensis* Franch.	Control of bacterial and viral infections, relax spasms, lower fevers, stimulate the circulation, treat diabetes mellitus, analgesic, locally anaesthetic, antibacterial, antipyretic, bitter, blood tonic, carminative, cholagogue, digestive, sedative, stomachic, vasodilator, treat diarrhoea, acute enteritis and dysentery, treat insomnia, fidget, delirium due to high fever, leukaemia and otitis media, treat conjunctivitis, skin problems (acne, boils, abscesses and burns whilst), mouth, tongue ulcers, swollen gums, and toothache	Uphof, 1959; Usher, 1974; Duke and Ayensu, 1985; Yeung, 1985; Bown, 1995
	*Coptis japonica* (Thunb.) Makino	Control of bacterial and viral infections, relax spasms, lower fevers, stimulate the circulation, locally analgesic and anaesthetic, anti-inflammatory, stomachic, treat conjunctivitis, intestinal catarrh, dysentery, enteritis, high fevers, inflamed mouth and tongue	Kariyone and Koiso, 1971; Usher, 1974; Grieve, 1984; Bown, 1995
	*Coptis teeta* Wall.	Control of bacterial and viral infections, relaxes spasms, lowers fevers and stimulate the circulation, locally analgesic, anaesthetic, ophthalmic and pectoral diseases, effective antibacterial, treat dysentery	Stuart and Smith, 1977; Duke and Ayensu, 1985; Bown, 1995
	*Hydrastis canadensis* L.	Treat disorders of the digestive system and mucous membranes, treat constipation, antiperiodic, antiseptic, astringent, cholagogue, diuretic, laxative, stomachic, tonic, treat disorders affecting the ears, eyes, throat, nose, stomach, intestines, and vagina	Uphof, 1959; Weiner, 1980; Grieve, 1984; Mills, 1985; Foster and Duke, 1990; Coffey, 1993; Bown, 1995; Chevallier, 1996; Lust, 2014
	*Xanthorhiza simplicissima* Marshall	Treat mouth ulcers, stomach ulcers, colds, jaundice, treat piles, and digestive disorders	Weiner, 1980; Foster and Duke, 1990; Moerman, 1998
Rutaceae	*Phellodendron amurense* Rupr.	Treat gastroenteritis, abdominal pain and diarrhea, antiinflammator, immunostimulator and treat cancer (antitumor activities)	Uchiyama et al., 1989; Park et al., 1999
	*Phellodendron chinense* C. K. Schneid. *Phellodendron chinense* var. *glabriusculum* C. K. Schneid. (ex-*Phellodendron wilsonii* Hayata & Kaneh.)	Act strongly on the kidneys, detoxicant for hot damp conditions, treat meningitis, conjunctivitis, antibacterial, antirheumatic, aphrodisiac, bitter stomachic, cholagogue, diuretic, expectorant, febrifuge, hypoglycaemic, treat ophtalmia, skin, vasodilator and tonic, treat acute diarrhoea, dysentery, jaundice, vaginal infections (with *Trichomonas vaginalis*), acute urinary tract infections, enteritis, boils, abscesses, night sweats and skin diseases, and expectorant	Kariyone and Koiso, 1971; Usher, 1974; Stuart and Smith, 1977; Grieve, 1984; Yeung, 1985; Bown, 1995; Chevallier, 1996
	*Zanthoxylum monophyllum* Tul.	Treat eye infections and dark vomitus	Hirschhorn, 1981; Eric Brussell, 2004

## Botanical sources of berberine

Berberine has been detected, isolated, and quantified from various plant families and genera including Annonaceae (*Annickia, Coelocline, Rollinia*, and *Xylopia*), Berberidaceae (*Berberis, Caulophyllum, Jeffersonia, Mahonia, Nandina*, and *Sinopodophyllum*), Menispermaceae (*Tinospora*), Papaveraceae (*Argemone, Bocconia, Chelidonium, Corydalis, Eschscholzia, Glaucium, Hunnemannia, Macleaya, Papaver*, and *Sanguinaria*), Ranunculaceae (*Coptis, Hydrastis*, and *Xanthorhiza*), and Rutaceae (*Evodia, Phellodendron*, and *Zanthoxyllum*) (Table 2). The genus *Berberis* is well-known as the most widely distributed natural source of berberine. The bark of *B. vulgaris* contains more than 8% of alkaloids, berberine being the major alkaloid (about 5%) (Arayne et al., 2007).

**Table 2:** Botanical sources of berberine.

**Family**	**Scientific name**	**Common name**	**Used part**	**References**
Annonaceae	*Annickia chlorantha* (Oliv.) Setten & Maas (ex-*Enantia chlorantha* Oliv.)	African whitewood, african yellow wood Epfoué, Péyé, Nfol, Poyo	Bark	Mell, 1929
	*Annickia pilosa* (Exell) Setten & Maas (ex-*Enantia pilosa* Exell)	–	Bark	Buzas and Egnell, 1965
	*Annickia polycarpa* (DC.) Setten & Maas ex I. M. Turner (ex-*Enantia polycarpa* (DC.) Engl. & Diels)	African yellow wood	Bark	Buzas and Egnell, 1965
	*Coelocline polycarpa* A.DC.	Yellow-dye tree of Soudan	Bark	Henry, 1949
	*Rollinia mucosa* (Jacq.) Baill.	Biriba, wild sweet sop, wild cashina	Fruit	Chen et al., 1996
	*Xylopia macrocarpa* A.Chev.	Jangkang	Stem bark	Willaman and Schubert, 1961
	*Xylopia polycarpa* (DC.) Oliv.	–	Stem bark	Willaman and Schubert, 1961
Berberidaceae	*Berberis aetnensis* C.Presl	–	Roots	Bonesi et al., 2013
			Leaves	Musumeci et al., 2003
			Root	Henry, 1949
	*Berberis amurensis* Rupr.	Barberry	Stem & roots	Tomita and Kugo, 1956
	*Berberis aquifolium* Pursh	Oregon grape	Roots	Parsons, 1882
	*Berberis aristata* DC.	Tree turmeric	Bark	Chakravarti et al., 1950
			Roots	Singh A. et al., 2010
			Stem	
			Raw herb	Singh R. et al., 2010
			Extract	
			Fruit	Kamal et al., 2011
			Roots	Andola et al., 2010a,c
			Roots	Rashmi et al., 2009
			Roots	Singh and Kakkar, 2009
			Roots	Srivastava et al., 2004
			Roots	Srivastava et al., 2001
			Bark	Willaman and Schubert, 1961
	*Berberis asiatica* Roxb. ex DC.	Chutro, rasanjan (Nep); marpyashi (Newa); daruharidra, darbi (Sans)	Roots	Andola et al., 2010b
			Roots	Andola et al., 2010c
			Roots	Srivastava et al., 2004
			Roots, stem, bark	Willaman and Schubert, 1961
	*Berberis barandana* Vidal.	–	ND	Willaman and Schubert, 1961
	*Berberis beaniana* C. K. Schneid.	Kang song xiao bo (pinyin, China)	–	Steffens et al., 1985
	*Berberis chitria* Buch.-Ham. ex Lindl.	Chitra, indian barberry	Whole plant	Hussaini and Shoeb, 1985
			Roots	Srivastava et al., 2006a,b,c
	*Berberis concinna* Hook.f.	Barberry	Stem bark	Tiwari and Masood, 1979
	*Berberis congestiflora* Gay	Michay	Leaves and stem	Torres et al., 1992
	*Berberis coriaria* Royle ex Lindl.	–	Stem bark	Tiwari and Masood, 1979
	*Berberis croatica* Mart. ex Schult. & Schult.f.	Croatian barberry	Roots	Končić et al., 2010
			Roots	Kosalec et al., 2009
	*Berberis darwinii* Hook.	Michai, calafate	Roots	Richert, 1918
			Leaves	Urzúa et al., 1984
			Stem-bark	Habtemariam, 2011
	*Berberis densiflora* Raf.	–	Leaves	Khamidov et al., 1997b
	*Berberis floribunda* Wall. ex G.Don	Nepal barberry	Roots	Chatterjee, 1951
	*Berberis fortunei* Lindl.	Fortune's Mahonia	Wood	Willaman and Schubert, 1961
	*Berberis guimpelii* K. Koch & C. D. Bouché	–	Roots	Petcu, 1965a
	*Berberis heteropoda* Schrank	–	Root bark	Willaman and Schubert, 1961
	*Berberis himalaica* Ahrendt	–	Stem-bark	Chatterjee et al., 1952
	*Berberis horrida* Gay	–	Leaves and stem	Torres et al., 1992
	*Berberis iliensis* Popov	–	Young shoots	Karimov and Shakirov, 1993
			Roots	Dzhalilov et al., 1963
	*Berberis integerrima* Bunge.	–	Root	Karimov et al., 1993
			Leaves	Karimov et al., 1993; Khamidov et al., 1996c, 1997b
	*Berberis jaeschkeana* C. K. Schneid.	Jaeschke's Barberry	–	Rashid and Malik, 1972
	*Berberis jamesonii* Lindl (ex-*Berberis glauca* Benth)	–	–	Willaman and Schubert, 1961
	*Berberis japonica* R.Br	Japanese Mahonia	Wood, root	Willaman and Schubert, 1961
	*Berberis kawakamii* Hayata	–	Roots	Yang and Lu, 1960a
	*Berberis koreana* Palib.	Korean barberry	Bark of the stem	Petcu, 1965b
			Bark of the roots	
			Seeds	
			Stem	
			Roots	
			–	Kostalova et al., 1982
			Roots	Yoo et al., 1986
			Leaves	
	*Berberis lambertii* R. Parker	–	Roots	Chatterjee and Banerjee, 1953
	*Berberis laurina* Thunb	Laurel barberry	Roots	Gurguel et al., 1934; Willaman and Schubert, 1961
	*Berberis leschenaultii* Wall. ex Wight & Arn (ex-*Mahonia leschenaultii* (Wall. ex Wight & Arn.) Takeda)	–	Bark	Willaman and Schubert, 1961
	*Berberis libanotica* Ehrenb. ex C. K. Schneid.	–	Root	Bonesi et al., 2013
	*Berberis lycium* Royle	Boxthorn barberry	Roots	Andola et al., 2010c
	*Berberis microphylla G. Forst*. (ex-*Berberis heterophylla* Juss. ex Poir. *Berberis buxifolia* Lam.)	Patagonian barberry, magellan barberry, calafate	Roots	Freile et al., 2006
			–	Rashid and Malik, 1972
	*Berberis mingetsensis* Hayata	–	Roots	Yang and Lu, 1960b
	*Berberis nummularia* Bunge	Nummular barberry	Young shoots	Karimov et al., 1993
	*Berberis morrisonensis* Hayata	–	Roots	Yang, 1960a,b
			Stem	
			–	
	*Berberis nepalensis* Spreng. (ex-*Mahonia acanthifolia* Wall. ex G.Don)	–	–	Willaman and Schubert, 1961
	*Berberis nervosa* Pursh	Dwarf Oregon-grape	–	Willaman and Schubert, 1961
	*Berberis oblonga* (Regel) C. K. Schneid	Oblong barberry	Stem	Karimov and Lutfullin, 1986; Gorval' and Grishkovets, 1999
			Leaves	Khamidov et al., 2003
			Roots	Tadzhibaev et al., 1974
	*Berberis petiolaris* Wall. ex G. Don	Chochar	Roots	Huq and Ikram, 1968
	*Berberis pseudumbellata* R. Parker	–	Roots	Andola et al., 2010b
			Stem bark	
			–	Pant et al., 1986
	*Berberis repens* Lindl.	Creeping mahonia, creeping Oregon grape, creeping barberry, or prostrate barberry	–	Willaman and Schubert, 1961
	*Berberis sargentiana* C. K. Schneid.	Sankezhen	–	Liu, 1992
	*Berberis swaseyi* Buckley	–	–	Willaman and Schubert, 1961
	*Berberis thunbergii* DC.	Japanese barberry	Stem	Khamidov et al., 1997a
			Leaves	Khamidov et al., 1997a
	*Berberis tinctoria* Lesch.	Nilgiri barberry	Roots	Srivastava and Rawat, 2007
	*Berberis trifolia* (Cham. & Schltdl.) Schult. & Schult.f.	–	Root, stem	Willaman and Schubert, 1961
	*Berberis turcomanica* Kar. ex Ledeb.	–	Leaves	Khamidov et al., 1996a,b,c
	*Berberis umbellata* Wall. ex G.Don	Himalayan barberry	Roots	Singh et al., 2012
	*Berberis vulgaris* L.	Barberry	Stems and roots	Imanshahidi and Hosseinzadeh, 2008
			Roots	Končić et al., 2010
			Roots	Kosalec et al., 2009
	*Berberis waziristanica* Hieron.	–	Root bark	Atta-ur-Rahma and Ahmad, 1992
	*Caulophyllum thalictroides* (L.) Michaux (ex-*Leontice thalictroides* L.)	Blue cohosh	–	Willaman and Schubert, 1961
	*Jeffersonia diphylla* (L.) Pers.	Twinleaf	–	Willaman and Schubert, 1961
	*Mahonia borealis* Takeda	–	–	Willaman and Schubert, 1961
	*Mahonia fortunei* (Lindl.) Fedde	Fortune's Mahonia	wood	Willaman and Schubert, 1961
	*Mahonia napaulensis* DC. (ex- *Mahonia griffithii*; ex-*Mahonia manipurensis* Takeda; *Mahonia sikkimensis* Takeda)	Nepal Barberry	bark	Willaman and Schubert, 1961
	*Mahonia simonsii* Takeda	–	–	Willaman and Schubert, 1961
	*Nandina domestica* Thunb.	Nandina, heavenly bamboo or sacred bamboo	bark, root	Willaman and Schubert, 1961
	*Sinopodophyllum hexandrum* (Royle) T.S.Ying	Himalayan May Apple, Indian may apple	Root, rhizome	Willaman and Schubert, 1961
Menispermaceae	*Tinospora sinensis* (Lour.) Merr*. (ex-Tinospora cordifolia)* (Willd.) Miers	Gulbel, indian tinospora	Stem	Srinivasan et al., 2008
			–	Singh et al., 2003
Papaveraceae	*Argemone albiflora* Hornem. (ex-*Argemone alba* F.Lestib.)	White prickly poppy, Bluestem pricklypoppy	Aerial part and roots	Slavikova et al., 1960
				Foote, 1932
				Israilov and Yunusov, 1986
	*Argemone hybrida* R.Otto & Verloove	–	Leaves and stem	Israilov and Yunusov, 1986
	*Argemone mexicana* L.	Prickly poppy	Apigeal parts, seeds	Haisova and Slavik, 1975; Israilov and Yunusov, 1986; Fletcher et al., 1993
			Leaves	Bapna et al., 2015
			Seeds	Fletcher et al., 1993
			–	Singh, 2014
			–	Majumder et al., 1956; Hakim et al., 1961; Misra et al., 1961
			Superterranean parts	Slavikova and Slavik, 1955
			Roots	
			–	Santos and Adkilen, 1932; de Almeida Costa, 1935; Misra et al., 1961; Doepke et al., 1976; Abou-Donia and El-Din, 1986; Monforte-Gonzalez et al., 2012
			Roots	Pathak et al., 1985; Kukula-Koch and Mroczek, 2015
			Leaves and capsules	Schlotterbeck, 1902
			Whole plant	Bose et al., 1963; Haisova and Slavik, 1975
			Latex	Santra and Saoji, 1971
	*Argemone ochroleuca* Sweet	Chicalote	Seeds	Fletcher et al., 1993
	*Argemone platyceras* L.	Chicalote poppy, crested poppy	Leaves and stem	Israilov and Yunusov, 1986
	*Argemone subintegrifolia* Ownbey	–	Aerial part	Stermitz et al., 1974
	*Argemone squarrosa* Greene	Hedgehog pricklypoppy	Aerial part	Stermitz, 1967
	*Bocconia frutescens* L.	Plume poppy, tree poppy, tree celandine, parrotweed, sea oxeye daisy, john crow bush	Leaves	Slavik and Slavikova, 1975
			Roots, stalks, leaves	Taborska et al., 1980
	*Chelidonium majus* L.	Celandine poppy	Roots	Jusiak, 1967
	*Corydalis chaerophylla* DC.	Fitweed	Roots	Jha et al., 2009
	*Corydalis ophiocarpa* Hook. f. *et* Thoms	Fitweed		Manske, 1939
	*Corydalis solida* subsp. *brachyloba*	Fitweed	Aerial parts	Sener and Temizer, 1988, 1991
	*Corydalis solida* subsp. *slivenensis* (Velen.) Hayek (ex-*Corydalis slivenensis* Velen.)	Fitweed	–	Kiryakov et al., 1982a,b
	*Corydalis solida* subsp. *tauricola*	Fitweed	–	Kiryakov et al., 1982b
			Rhizome	Sener and Temizer, 1990
	*Corydalis turtschaninovii* Besser. (ex-*Corydalis ternata* (Nakai) Nakai)	Fitweed	Tubers	Lee and Kim, 1999
	*Eschscholzia californica* Cham.	Californian poppy	Roots	Gertig, 1964
	*Glaucium corniculatum* (L.) Rud. subsp. *corniculatum*	Blackspot Hornpoppy	Aerial parts	Doncheva et al., 2014
			–	Slavik and Slavikova, 1957
	*Glaucium grandiflorum* Boiss. & A.Huet	Red Horned Poppy, Grand-flowered Horned Poppy	Aerial part	Phillipson et al., 1981
	*Hunnemannia fumariifolia* Sweet	Mexican Tulip Poppy, Golden Cup	Roots	Slavikova and Slavik, 1966
	*Macleaya cordata* (Willd.) R.Br.	Plume poppy	–	Kosina et al., 2010
	*Macleaya microcarpa* (Maxim.) Fedde	Poppy	Roots	Pěnčíková et al., 2011
	*Papaver dubium* L.	Long-Head Poppy	Roots	Slavik et al., 1989
	*Papaver dubium var. lecoquii*	Long-Head Poppy	Latex	Egels, 1959
	*Papaver rhoeas* L. var. *chelidonioides*	Corn Poppy	Roots	Slavík, 1978
	*Papaver hybridum* L.	Poppy	Aerial part	Phillipson et al., 1981
	*Sanguinaria canadensis* L.	Bloodroot		Greathouse, 1939
Ranunculaceae	*Coptis chinensis* Franch.	Chinese goldthread	Roots	Jin and Shan, 1982
			Roots	Lou et al., 1982
	*Coptis japonica* (Thunb.) Makino	Japanese goldthread	Rhizome	Kubota et al., 1980
	*Coptis teeta* Wall.	Gold thread	Rhizome	Chen and Chen, 1988
			Rhizome	Zhang et al., 2008
			Roots	
	*Hydrastis canadensis* L.	Goldenseal	–	Baldazzi et al., 1998
			–	Leone et al., 1996
	*Xanthorhiza simplicissima* Marshall	Yellowroot	Root, stem, and leaves	Okunade et al., 1994
Rutaceae	*Evodia meliaefolia (*Hance ex Walp.) Benth.	–	Bark	Perkin and Hummel, 1895
	*Phellodendron amurense* Rupr.	Amur cork tree	Bark	Chiang et al., 2006
			Root bark	Zhang et al., 2008
			Trunk bark	
			Perennial Branch bark	
			Annual branches	
			Leaves	
	*Phellodendron chinense* C. K. Schneid.	Chinese cork tree	Bark	Chan et al., 2007
	*Phellodendron chinense* var. *glabriusculum* C. K. Schneid. (ex-*Phellodendron wilsonii* Hayata & Kaneh.)	Chinese cork tree	Bark, branch, leaf and heartwood	Chen, 1981
			–	Tan et al., 2013
			Bark	Chen, 1982
	*Phellodendron lavallei* Dode	Lavalle corktree	Bark	Yavich et al., 1993
	*Zanthoxylum monophyllum* (Lam.) P. Wilson	Palo rubio	Stem and branches	Stermitz and Sharifi, 1977
	*Zanthoxylum quinduense* Tul.	–	–	Ladino and Suárez, 2010

Berberine is also widely present in barks, leaves, twigs, rhizomes, roots, and stems of several medicinal plants species, including *Argemone mexicana* (Etminan et al., 2005), *Berberis aristata, B. aquifolium, B. heterophylla, B. beaniana, Coscinium fenestratum* (Rojsanga and Gritsanapan, 2005), *C. chinensis, C. japonica, C. rhizome, Hydratis canadensis* (Imanshahidi and Hosseinzadeh, 2008), *Phellodendron amurense, P. chinense, Tinospora cordifolia* (Khan et al., 2011), *Xanthorhiza simplicissima* (Bose et al., 1963; Knapp et al., 1967; Sato and Yamada, 1984; Steffens et al., 1985; Inbaraj et al., 2001; Liu et al., 2008a; Srinivasan et al., 2008; Vuddanda et al., 2010). Several researches found that berberine is widely distributed in the barks, roots, and stems of plants, nevertheless, bark and roots are richer in berberine compared to other plant parts (Andola et al., 2010a,b). In the Papaveraceae family, *Chelidonium majus* is another important herbal source of berberine (Tomè and Colombo, 1995). An important number of plants for medicinal use, such as *Coptidis rhizoma* and barberry, are the natural sources with the highest concentration of berberine. Barberries, such as *Berberis aristata, B. aquifolium, B. asiatica, B. croatica, B. thunbergii*, and *B. vulgaris*, are shrubs grown mainly in Asia and Europe, and their barks, fruits, leaves, and roots are often widely used as folk medicines (Imanshahidi and Hosseinzadeh, 2008; Kosalec et al., 2009; Andola et al., 2010c; Kulkarni and Dhir, 2010). Different research groups have reported that maximum berberine concentration accumulates in root (1.6–4.3%) and in most of the *Berberis* species, plants that grow at low altitude contain more berberine compared to higher altitude plants (Chandra and Purohit, 1980; Mikage and Mouri, 1999; Andola et al., 2010a). However, a correlation could not be established within the results of berberine concentration regarding to species and season of the year (Srivastava et al., 2006a,c; Andola et al., 2010c; Singh et al., 2012). Comparative studies of berberine concentration contained in different species of the same genus have been reported, e.g., higher berberine content in *B. asiatica* (4.3%) in comparison to *B. lycium* (4.0%), and *B. aristata* (3.8%). Meanwhile, Srivastava et al. (2004) documented a higher berberine content in root of *B. aristata* (2.8%) compared with *B. asiatica* (2.4%) (Andola et al., 2010a). Seasonal variation of berberine concentration has been reported, e.g., the maximum yield of berberine for *B. pseudumbellata* was obtained in the summer harvest, and was 2.8% in the roots and 1.8% for the stem bark, contrary to that reported in the roots of *B. aristata*, where the berberine concentration (1.9%) is higher for the winter harvest (Rashmi et al., 2009). These variations may be caused to multiple factors, among which stand out: (i) the intraspecific differences, (ii) location and/or, (iii) the analytical techniques used. Table 2 gathers a synthesis of the main species containing berberine.

## Extraction methods

Berberine, a quaternary protoberberine alkaloid (QPA) is one of the most widely distributed alkaloid of its class. Current studies suggest that isolation of the QPA alkaloids from their matrix can be performed using several methods. The principles behind these methods consist of the interconversion reaction between the protoberberine salt and the base. The salts are soluble in water, stable in acidic, and neutral media, while the base is soluble in organic solvents. Thus during the extraction procedure, the protoberberine salts are converted in their specific bases and further extracted in the organic solvents (Marek et al., 2003; Grycová et al., 2007).

In the case of berberine, the classical extraction techniques like maceration, percolation, Soxhlet, cold or hot continuous extraction are using different solvent systems like methanol, ethanol, chloroform, aqueous, and/or acidified mixtures. Berberine's sensitivity to light and heat is the major challenge for its extraction. Hence, exposure to high temperature and light could lead to berberine degradation and thus influencing its matrix recovery. In his study Babu et al. (2012) demonstrated that temperature represent a crucial factor in both extraction and drying treatments prior extraction. The yield of berberine content in *C. fenestratum* stem tissue samples was higher in case of samples dried under the constant shade with 4.6% weight/weight (*w/w*) as compared to samples dried in oven at 65°C (1.32% *w/w*) or sun drying (3.21% *w/w*). As well hot extraction procedure with methanol or ethanol at 50°C gave lower extraction yields when compared with methanol or ethanol cold extraction at −20°C. Thus, berberine content in the shade-dried samples was 4.6% (*w/w*) for methanolic cold extraction and 1.29% (*w/w*) for methanolic hot extraction (Babu et al., 2012).

**Figure 2:**
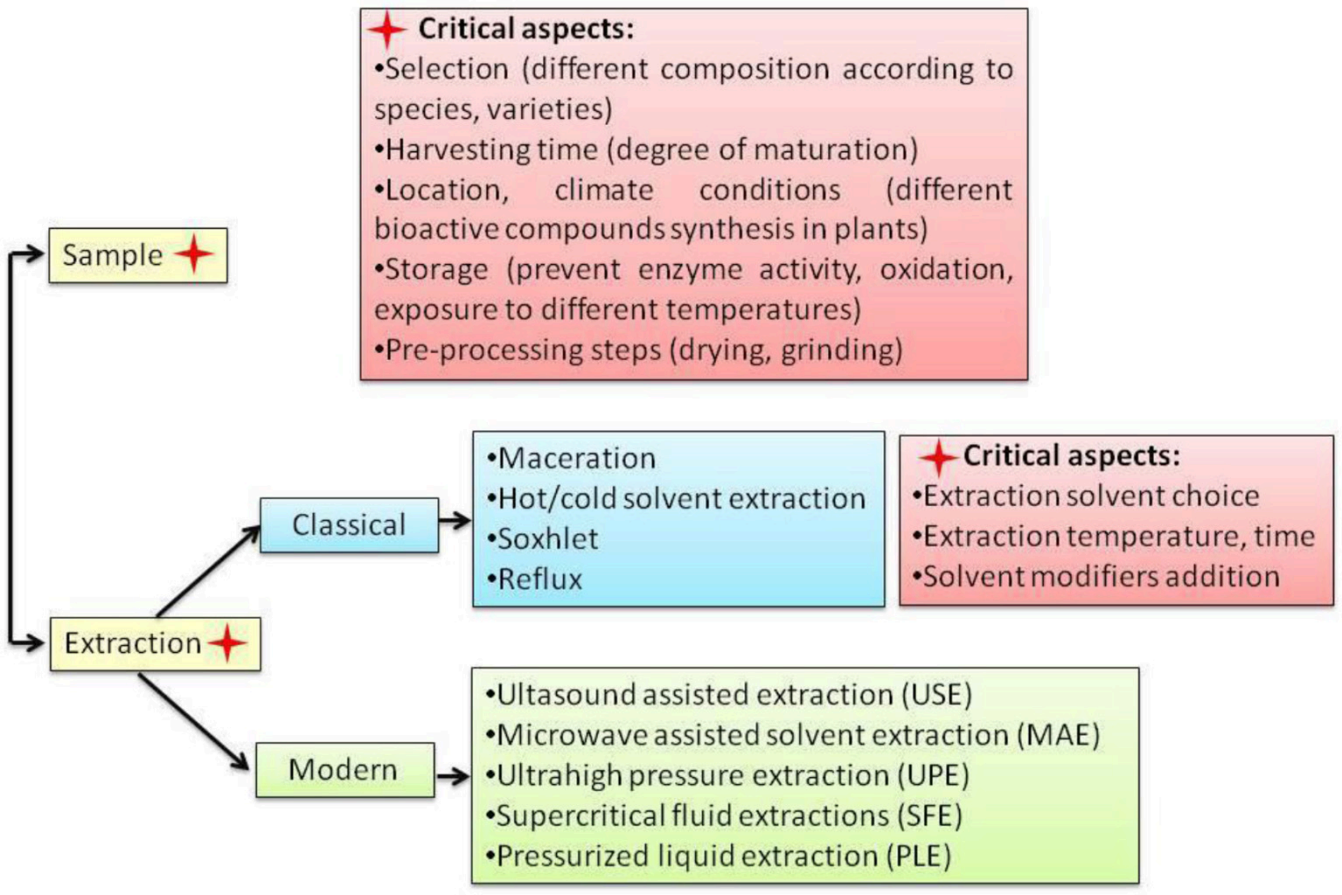
Short view on berberine extraction methods.

Along with extraction temperature, the choice of solvents is considered a critical step in berberine extraction as well (Figure 2). As seen in Table 3, methanol, ethanol, aqueous or acidified methanol or ethanol are the most used extraction solvents. The acidified solvents (usually with the addition of 0.5% of inorganic or organic acids) are used to combine with free base organic alkaloids and transform them in alkaloid salts with higher solubility (Teng and Choi, 2013). The effect of different inorganic acids like hydrochloric acid, phosphoric acid, nitric acid, and sulfuric acid as well as the effect of an organic acid like acetic acid were tested on berberine content and other alkaloids in rhizomes of *Coptis chinensis* Franch by Teng and Choi (2013). In this case, 0.34% phosphoric acid concentration was considered optimal. Moreover, when compared to other classical extraction techniques like reflux and Soxhlet extraction, the cold acid assisted extraction gave 1.1 times higher berberine yields.

**Table 3:** Extraction and detection methods for berberine in different herbal and biological matrixes.

**Sample (weight)**	**Extraction method**	**Detection method**	**References**
Dried stem powder *Coscinium fenestratum* (Gaertn.) (1 g)	**Extraction solvents (ES):** water, methanol–water (1:1. *v*/*v*), and methanol **Sonication** (15 min, room temperature) Centrifugation (2,800 rpm, 15 min) Filtration and evaporation Extracts resolubilization (methanol:water, 9:1 *v*/*v*)	**HPLC - DAD** Column: ODS, Chromolith, RP-18e,100 × 4.6 mm Mobile Phases: Methanol/Deionized Water (90:10, *v*/*v*) Flow: 0.5 mL/min, Temperature: 25°C **UV Spectrophotometric Analysis**	Akowuah et al., 2014
*C. fenestratum* (Gaertn.) (10 g)	**ES:** methanol **Hot extraction:** sample refluxed with ES for 3 h Filtration and evaporation. Extracts resolubilization (methanol)	**TLC** Adsorbent: Silica Gel GF _254_ Solvent system: n-Butanol: Ethyl acetate: Acetic acid (2.5:1.5:1, *v*/*v/v*) Detection: 254 and 366 nm	Arawwawala and Wickramaar, 2012
	**Cold extraction:** sample extraction with ES for 24 h Filtration and evaporation. Extracts resolubilization (methanol)		
Dried C. *fenestratum* (0.1 g)	**ES**: absolute methanol **Cold extraction:** sample extraction at −20°C **Hot extraction:** water bath sample extraction at 50°C **ES**: absolute ethanol **Cold extraction** sample extraction at −20°C **Hot extraction:** water bath sample extraction at 50°C Samples centrifugation (10 min at 10°C after cooling down) Samples filtration	**HPLC** Column: C18, 250 × 4.6 mm, 5 μm Mobile Phases: Acetonitrile/0.1% Trifluro-acetic acid (50:50, *v*/*v*) Detection: 344 nm Flow: 0.8 mL/min	Babu et al., 2012
*C. fenestratum* (1,000g) *Capsules* (containing 62.5 mg *C. fenestratum*)	**ES**: petroleum ether, chloroform, methanol (1L each) **Soxhlet extraction**: with each ES for 3 days at (30–40°C) **ES:** methanol (10 mL) Extraction for 1 h Filtration and evaporation Resolubilisation in methanol (5 mL)	**HPLC** Column: Luna C18, 150 × 4.6 mm, 5 μm, Phenomenex Mobile Phases: (A) Potassium dihydrogen phosphate (pH −2.5) and (B) Acetonitrile Detection: 220 nm Flow: 1 mL/min **HPTLC** Adsorbent: Silica Gel 60F _254_ Solvent system: n-Butanol: Glacial acetic acid: Water (8:1:1, *v*/*v/v*) Detection: 350 nm for all measurements	Jayaprakasam and Ravi, 2014
*Tinospora cordifolia* (Willd.), *Tribulus terrestris* (L.), *Emblica officinalis* (Gaertn.) (3 g)	**ES**: chloroform Dried sample trituration with ammonia solution Drying at room temperature Extraction with ES for 1h Chloroform phase extraction with 5% sulfuric acid (x 3) Basification of acid extract with sodium carbonate (pH −9) Extraction of basified solution with chloroform (X 3) Evaporation of chloroform phase (temperature under 50°C) Residue solubilization with methanol	**UV-VIS** UV absorbance: 348 nm	Joshi and Kanaki, 2013
*Cortex phellodendri* (2 g)	**Ultrahigh pressure extraction (UPE)** Optimal parameters: ES: ethanol (69.1%), liquid-solid ratio−31.3, extracting pressure−243.30 MPa, extraction time−2 min	**HPLC** Column: Hypersil ODS C18, 250 × 4.6 mm, 5 μm Mobile Phases: (A) 0.3% triethanolamine aqueous solution (pH − 3.5) Detection: 265 nm Temperature: 30°C Flow: 1 mL/min	Guoping et al., 2012
Rhizome of *Coptis chinensis* Franch (1 g)	**Supercritical fluid extraction** Extraction time: up to 3 h Temperature: 60°C Pressure: from 200 to 500 bar Flow-rate of carbon dioxide (gaseous state): 1 L/min Flow-rate of modifier: 0.4 mL/min. Organic solvent modifier systems: ethanol-modified supercritical carbon dioxide, methanol-modified supercritical carbon dioxide, 1,2-propanediol-modified supercritical carbon dioxide, 5% Tween 80 in methanol-modified supercritical carbon dioxide, 5% Tween in ethanol-modified supercritical carbon dioxide	**HPLC** Column: Diamonsil C18, 250 × 4.6 mm, 5 μm Mobile Phases: 33 mM Potassium dihydrogen phosphate : acetonitrile (70:30, *v/v*) Detection: 345 nm Flow: 1 mL/min	Liu et al., 2006
	**Soxhlet extraction** **ES**: hydrochloric acid: methanol (1: 100, *v/v*) Time: 8 h		
*Cortex pellodendri amurensis* (1 g)	**Ultrahigh pressure extraction** **ES**: ethanol (50 %), liquid-solid ratio −30: 1, extracting pressure −400 MPa, extraction time −4 min, extraction temperaturte −40°C **Ultrasonic extraction** **ES**: 70% ethanol Sample soaking for 24 h in 40 ml ES Sonic extraction for 60 min at 30°C	**HPLC- DAD** Column: Daisopak SP-120-5-ODS_BP, 250 × 4.6 mm, 5 μm Mobile Phases: (A) acetonitrile and (B) phosphoric acid: water (0.7:100, *v/v*) Detection: 345 nm Temperature: 25°C Flow: 1 mL/min	Liu et al., 2013
	**Heat reflux extraction** **ES**: 70% ethanol Sample soaking for 24 h in 40 ml ES Sample extraction for 4 h at boiling state		
	**Soxhlet extraction** **ES**: 70% ethanol Sample soaking for 24 h in 40 ml ES Sample extraction: 4 h		
Goldenseal *(Hydrastis canadensis* L.) (2, 5, 5 g)	**Pressurized hot water extraction** **ES:** water at 140°C, Optimal parameters: pressure: 50 bars and flow rate: 1 mL/min, Time: 15 min **Reflux extraction** **ES:** methanol (200 mL) Sonication: 4 h at 80°C **Ultrasonic extraction** **ES:** methanol (50 mL) Reflux: 6 h with continuous stirring	**HPLC-DAD** Column: Zorbax eclipse Plus C 18, 75 x 4.6 mm, 3.5 μm Mobile phases: (A) 0.1 % Formic Acid (pH 2.7) and (B) methanol Detection: 242 nm Temperature: 35°C Flow: 1 mL/min **MS** Detection: ESI (+) Capillary temperature: 200°C, Sheath gas: 80, Capillary voltage: 20 V, Tube lens voltage: 5V	Mokgadi et al., 2013
*Berberis aristata* DC (1.5 g), *Berberis aristata* herb extract (0.1 g), Ayurvedic form (6 g)	**Crude herb reflux extraction** **ES**: methanol (100 mL) for 1 h in a water bath Filtratio Reextraction with ES (50 mL) for 30 min (× 2) Filtrates combination and concentration to 50 mL **Herb extracts ultrasonic extraction** **ES**: methanol (up to 10 mL) Sonication Filtration	**HPLC** Column: Zorbax ODS II, 250 x 4.6 mm, 5 μm Mobile phase: potassium hydrogen phosphate buffer (pH 2.5)/ acetonitrile Detection: 346 nm Temperature: 40°C Flow: 1 mL/min	Singh R. et al., 2010
	**Ayrvedic form ultrasonic extraction** **ES**: methanol (up to 25 mL) Sonication		
*Berberis aristata* DC root	**Soxhlet extraction** **ES**: ethanol **Berberine isolation** Ethanolic extract concentration to obtain a syrup mass Dissolvation in hot water and filtration Acidification (36.5% w/v hydrochloric acid) Cool: ice bath - 30 min, overnight in refrigerator	**HPTLC** Stationary phase: precoated silica gel 60GF254 Mobile phases: n-butanol: glacial acetic acid: water (12:3:4 *v/v/v*) Temperature: 33 ± 5°C Detection: 350 nm	Patel, 2013
*Mahonia manipurensis* (Takeda) stem bark (100 g)	**Cold extraction** **ES**: 80% methanol (1,000 mL) Stirring at room temperature Extract concentration	**TLC** Stationary phase: precoated silica gel G F254 Mobile phase: hexane: ethyl acetate: methanol (56:20:5) Fraction purification: positive test using Dragendroff's reagent Further analysis of purified fraction Mobile phase: chloroform: ethyl acetate: diethylamine: methanol: 20% ammonium hidroxide (6:24:1.5:6:0.3)	Pfoze et al., 2014
		**HPLC** Column: Water Symmetry C18, 250 x 4.6 mm, 5 μm Mobile phase: methanol/ formic acid buffer (0.1%, *v/v*) Detection: 346 nm Flow: 1 mL/min	
		**UV-VIS** UV spectra: 200–500 nm	
		**ESI-MS**	
*Coscinium fenestratum* (100 g)	**Maceration** **ES:** 80% ethanol (500 ml), 160 h Shaken: 80 h (200 rpm), stand: 80 h Reextraction: 48 h, shaken: 24 h, stand: 24 h Combined extracts concentration Evaporation to dryness (dry extract) Resolubilisation in 80% ethanol (10 mg dry extract/mL)	**TLC** Stationary phase: Silica gel GF254 Mobile phase: ethyl acetate : butanol : formic acid : water (50:30:12:10); Detection: 366 nm	Rojsanga and Gritsanapan, 2005
*Argemone mexicana*	**Soxhlet extraction** **ES**: methanol Evaporation to dryness Resolubilisation in methanol (known concentration)	**HPTLC** Stationary phase: precoated silica gel 60F254 Mobile phases: toluene: ethyl acetate (9:3, *v/v*). Detection: 266 nm	Samal, 2013
*Tinospora cordifolia* (20 g)	**Microwave assisted extraction (MAE)** **ES**: 80% ethanol Irradiation power: 60%, Extraction time: 3 min **Soxhlet extraction** **ES**: ethanol, for 3 h Filtration Concentration	**HPTLC** Mobile phases: methanol: acetic acid: water (8: 1: 1, *v/v/v*). Detection: 366 nm	Satija et al., 2015
	**Maceration** **ES**: ethanol (200 mL), 7 days, occasional stirring		
*Berberis aristata, Berberis tinctoria* (800 g)	**Hot extraction** **ES**: methanol (2.5 L) (X2) Extraction time: 3 h Temperature: 50°C Extract concentration under vacuum	**HPLC** Column: Unisphere C18, 150 x 4.6 mm, 5 μm Mobile phase: (A) 0.1% trifluoroacetic acid and (B) acetonitrile (60:40, *v/v*) Detection: 350 nm Temperature: 30°C Flow: 1 mL/min	Shigwan et al., 2013
*Coptis chinensis* Franch. (1g)	**Acid assisted extraction** **ES**: several inorganic acids (hydrochloric acid, phosphoric acid, nitric acid, and sulfuric acid) and one organic acid (acetic acid) Extraction time:1–8 h, Acid concentrations: 0–1% Solvent to sample ratios: 20–60 mL/g Maceration at 25°C Filtration Dilution to 100 mL final volume	**HPLC** Column: XTerra C18, 250 x 4.6 mm Mobile phase: (A) acetonitrile and (B) 25 mmol/L potassium dihydrogen phosphate,(27:75, *v/v*) Detection: 345 nm Temperature: 30°C	Teng and Choi, 2013
	**Soxhlet extraction** **ES**: 50% ethanol (100 mL), 4 h at 70°C Extract evaporation to dryness Resolubilization in ES (up to 100 mL final volume)		
	**Heating reflux extraction** **ES**: 50% ethanol Soaked for 1 h Extraction: 4 h at 70°C (heated water bath) Filtration Dilution (up to 100 mL final volume)		
Rabbit plasma (100 μl)	Mixing 100 μl sample with 3% formic acid in acetonitrile (200 μl) Vortex: 30 s Centrifugation: 10 min at 4°C Evaporation of supernatant: under nitrogen stream at 40°C	**LC-ESI-MS** HPLC system Column: Capcell Pakc_18_ MG, 100 × 2.1 mm, 5 μm with Security Guard C18, 4 × 2 mm, 5 μm Mobile Phases: (A) 0.4% formic acid solution and (B) 0.2 % formic acid solution in methanol (60:40, *v/v*)	Liu et al., 2011
	Residue solubilization: in 100 μl of 20% methanol	Temperature: 25°C Flow: 0.4 mL/min MS detection: Source: ESI (+) Quantification: MRM mode	
Rat plasma	**Solid phase extraction (SPE)** Cartridges: Oasis HLB (1 cc, 30 mg) Pre-conditioning: 2 mL methanol Equilibrtating:	**UPLC-MS/MS** UPLC system Column: 120 EC-C18, 50 × 4.6 mm, 2.7 μm with Security Guard C18, 4 × 2 mm, 5 μm Mobile Phases: (A) 10 mM ammonium acetate in water (pH- 4.5) and (B) acetonitrile Temperature: 35°C Flow: 0.8 mL/min MS detection: Source: ESI (+) Quantification: MRM mode	Liu M. et al., 2015
Rat plasma Rat tissue	**Rat plasma** **ES**: methanol Mixing sample (200 μl) with internal standard (40 μl) and ES (560 μl) Vortex: 20 s Centrifugation: 10 min, 12,000x *g* Filtration	**UPLC-MS/MS** UPLC system Column: Acquity BEH C18, 50 × 2.1 mm, 1.7 μm Mobile Phases: (A) acetonitrile and (B) formic acid: water (0.1:99.9, *v/v*) Flow: 0.25 mL/min MS detection: Source: ESI (+) Quantification: MRM mode	Wang et al., 2016
	**Rat tissue** Grinding: 3 mL physiological saline with 600 mg tissue Centrifugation: 10 min, 12,000x *g*, 4°C Mixing supernatant (200 μl) with internal standard (40 μl) and ES (560 μl) Vortex: 20 s Centrifugation: 10 min, 12,000x *g* Filtration		
Rat plasma	Evaporation of 10 ul IS in the working tube Mixing sample (200 μl) with internal evaporated standard Vortex: 1 min Mixing sample with 10 μl 1% formic acid and 200 μl acetone Vortex: 2 min Centrifugation: 10 min, 10,000 rpm Mixing supernatant with 200 μl methanol Vortexing, centrifugation Mixing supernatant wit 400 μl acetonitrile Vortexing, centrifugation Evaporation to dryness (37°C, under nitrogen stream) Resolubilization in methanol	**LC-MS/MS** LC system Column: Zorbax Eclipse XDB-C18, 150 × 2.1 mm, 3.5 μm Mobile Phases: (A) acetonitrile and (B) water with 1% acetic acid and 0.001 mol/L ammonium acetate Flow: 0.2 mL/min MS detection: Source: ESI (+) Quantification: MRM mode	Xu et al., 2015
Rat plasma	**ES**: 90% methanol Mixing sample (100 μl) with internal standard (10 μl) and ES (100 μl) Vortex: 1 min Centrifugation: 10 min, 12,000 rpm, 4°C Supernatant evaporation to dryness under nitrogen stream Resolubilization (100 μl ES)	**UPLC-MS/MS** UPLC system Column: Acquity UPLC BEH C18, 50 × 2.1 mm, 1.7 μm Mobile Phases: (A) formic acid: water (0.1:99.9, *v/v*) and (B) acetonitrile Flow: 0.4 mL/min MS detection: Source: ESI Quantification: MRM mode	Yang et al., 2017

Large solvent volumes and long extraction time represent other drawbacks of conventional extraction methods (Mokgadi et al., 2013). For example, Rojsanga and Gritsanapan (2005) used maceration process to extract 100 g of *C. fenestratum* plant material with a total volume of 3,200 mL solvent (80% ethanol) over a period of 416 h. Furthermore, in a different study, Rojsanga et al. (2006) used several classical extraction techniques like maceration, percolation, and Soxhlet extraction to extract the berberine from *C. fenestratum* stems. This time even if the extracted plant material was in a lower amount than the previous study (30 vs. 100 g), large solvent volumes (2,000 mL for maceration, 5,000 mL for percolation, and 600 mL for Soxhlet extraction) over long time periods (7 days for maceration and 72 h for Soxhlet extraction) were employed (Rojsanga and Gritsanapan, 2005; Rojsanga et al., 2006).

Large solvent volumes are characteristic for other conventional methods too. Shigwan et al. (2013) extracted berberine from *Berberis aristata* and *B. tinctoria* powdered stem bark (800 g) using hot extraction (50°C for 3 h) with 2,500 mL methanol (Shigwan et al., 2013).

Even though conventional methods are widely used in berberine extraction, a number of other different methods have been developed lately. This led to an improved extraction efficiency, a decreased extraction time and solvents' volumes used in the extraction. Thus, ultrasound assisted solvent extraction (USE), microwave-assisted solvent extraction (MAE), ultrahigh pressure extraction (UPE), and supercritical fluid extractions (SFE), pressurized liquid extraction (PLE) have been successfully used as alternative extraction techniques with better results when compared with classical extraction methods.

Ultrasonically and microwave-assisted extraction are considered green, simple, efficient, and inexpensive techniques (Alupului et al., 2009).

Teng and Choi (2013) extracted berberine from *Rhizome coptidis* by optimized USE. Using response surface methodology, they identified that the optimal extraction conditions were 59% ethanol concentration, at 66.22°C within 46.57 min. A decrease in the extraction time (39.81 min) was obtained by Chang (2013). He used the combination of ionic liquids solutions as green solvents with USE to extract berberine from *Coptis chinensis* in order to apply an environmentally friendly approach (Chang, 2013). Moreover, in their study, Xu et al. (2017) compared several extraction tehniques like USE, distillation, and Soxhlet extraction in order to establish an high-efficient method for phellodendrine, berberine, and palmatine extraction from fresh *Phellodendron* bark (*Cortex phellodendri*). In the case of berberine, the combination of simple or acidified solvent (water, ethanol, and methanol) with the adjustment of the specific setting characteristics to each extraction type enabled them to determine the highest extraction yield. They concluded that the use of USE and hydrochloric acid-acidified methanol were the most efficient in extracting berberine. The USE extraction yield was significantly higher when compared to distillation and Soxhlet extraction, with values of ~100 mg/g toward 50 and 40 mg/g berberine, respectively (Xu et al., 2017).

The important reduction in organic solvent and extraction time determined the increasing interest in MAE, too. Lately, MAE was used as a green and cost-effective alternative to conventional methods. Using central composite design, Satija et al. (2015) successfully optimized the MAE parameters in terms of irradiation power, time, and solvent concentration to extract berberine form *Tinospora cardifolia*. They compared two classical extraction techniques like maceration and Soxhlet extraction with MAE under optimized conditions (60% irradiation power, 80% ethanol concentration, and 3 min extraction time). The results showed that MAE extraction had the highest yield of berberine content with 1.66% (*w/w*) while Soxhlet and maceration had 1.04 and 0.28% (*w/w*), respectively. Their study is emphasizing the dramatic time reduction in case of MAE (3 min) when compared with Soxhlet extraction (3 h) and maceration (7 days) together with solvent and energy consumption (Satija et al., 2015).

Another novel extraction technique considered to be environmentally friendly is UPE. The interest toward this extraction technique is increasing because it presents several advantages toward classical extraction techniques like increased extraction yields, higher quality of extracts, less extraction time, and decreased solvent consumption (Xi, 2015). These are achieved at room temperature by applying different pressure levels (from 100 to 600 MPa) between the interior (higher values) and the exterior of cells (lower values) in order to facilitate the transfer of the bioactive compounds through the plant matrices in the extraction solvent (Liu et al., 2006, 2013). In the study regarding berberine content in *Cortex phellodendri*, Guoping et al. (2012) made a comparison between UPE, MAE, USE, and heat reflux extraction techniques. They observed that the higher extraction yield and the lower extraction time was obtained in case of UPE with 7.7 mg/g and 2 min extraction time toward reflux, USE and MAE with 5.35 mg/g and 2 h, 5.61 mg/g and 1 h. and 6 mg/g and 15 min, respectively (Guoping et al., 2012).

Super critical fluid extraction is another environmentally friendly efficient technique used in phytochemical extraction. Because the extraction is performed in the absence of light and oxygen, the degradation of bioactive compounds is reduced. Also, the inert and non-toxic carbon dioxide used as a main extraction solvent in combination with various modifiers (e.g., methanol) and surfactants (e.g., Tween 80) at lower temperatures and relatively low pressure, allows the efficient extraction of bioactive compounds (Liu et al., 2006; Farías-Campomanes et al., 2015). In case of berberine extraction from the powdered rhizome of *Coptis chinensis* Franch, the highest recovery of berberine was obtained when 1,2-propanediol was used as a modifier of supercritical CO_2_ (Liu et al., 2006).

Pressurized liquid extraction, also known as pressurized fluid extraction, pressurized solvent extraction, and accelerated solvent extraction (ASE) is considered a green technology used for compounds extraction from plants (Mustafa and Turner, 2011). Compared with conventional methods, PLE increases the extraction yield, decreases time and solvent consumption, and protects sensitive compounds. In their study, Schieffer and Pfeiffer (2001) compared different extraction techniques like PLE, multiple USE, single USE, and Soxhlet extraction in order to extract berberine from goldenseal (*Hydrastis canadensis*). When compared in terms of extraction yield the results are comparable, ~42 mg/g berberine, except single USE with slightly lower content (37 mg/g berberine). Big differences were observed in the extraction time, PLE requiring only 30 min for a single sample extraction compared to 2 h for multiple extraction techniques or 6 h for Soxhlet extraction (Schieffer and Pfeiffer, 2001).

When referring to berberine extraction from biological samples, the extraction process is relatively simple and involves several steps like sample mixing with extraction solvents (e.g., methanol, acetone, acetonitrile), vortex, centrifugation followed by supernatant evaporation under nitrogen stream (Table 3). Other extraction techniques like solid phase extraction (SPE) can also be applied.

## Analytical techniques

After extraction and purification, the separation and quantification of berberine are commonly resolved by chromatographic methods. According to literature studies, berberine determination in plants was predominantly performed using methods like UV spectrophotometry (Joshi and Kanaki, 2013), HPLC (Babu et al., 2012; Akowuah et al., 2014), HPTLC and TLC (Rojsanga and Gritsanapan, 2005; Arawwawala and Wickramaar, 2012; Samal, 2013), capillary electrophoresis (Du and Wang, 2010), while berberine content in biological fluids was mainly achieved by using LC-MS (Deng et al., 2008; Feng et al., 2010), UPLC-MS (Liu M. et al., 2015; Liu L. et al., 2016), UHPLC/Q-TOF-MS (Wu et al., 2015).

UV-Vis spectrophotometry can be considered as one of the most rapid detection methods for berberine quantitative analysis from plant extracts. Based on the Beer-Lambert law, berberine concentration can be determined according to its absorption maxima at 348 nm. Joshi and Kanaki (2013) quantified berberine in *Rasayana churna* samples in the range of 2–20 μg/mL, the interference with other compounds being avoided by the specific isolation of the alkaloid fraction (Joshi and Kanaki, 2013).

Next, high-performance liquid chromatography (HPLC) is a versatile, robust, and widely used technique for the qualitative and quantitative analysis of natural products (Sasidharan et al., 2011). This approach is widely used in berberine identification and quantification. Generally, the choices of stationary phase in berberine separation are variants of C18-based silica column (Table 3) with a mobile phase consisting of simple or acidified solvents like water, methanol, or acetonitrile, used as such or in combination with phosphate buffers. Normally, the identification and separation of berberine can be accomplished using either isocratic or gradient elution system. Berberine identification is further accomplished using high sensitivity UV or DAD (diode array detectors) detectors. For example, Shigwan et al. (2013) developed in his study a reverse phase HPLC method with photodiode array detection (PDA) to quantify berberine from *Berberis aristata* and *B. tinctoria*. They used a Unisphere-C18 column (5 μm, 4.6 × 150 mm) with an isocratic gradient of acidified water (with 0.1% trifluoroacetic acid) and acetonitrile (60:40, *v/v*) to elute berberine within 5 min. The developed method was reproducible, validated, precise, and specific for berberine quantification (with a concentration range between 0.2 and 150 μg/mL; Shigwan et al., 2013).

Two other commonly used techniques in berberine quantification are thin layer chromatography (TLC) and high performance thin layer chromatography (HTPLC). Sometimes, these methods are preferred over HPLC, offering the possibility of running several samples simultaneously along with the use of small amount of both samples and mobile phases (Samal, 2013). For these reasons, Samal (2013) used an HPTLC method to quantify berberine from *A. mexicana* L. using toluene and ethyl acetate (9:3, *v/v*) as mobile phases, and a silica gel plate as stationary phase, they developed a simple, rapid, and cost-effective method for berberine quantification. The LOD (0.120 μg) and LOQ (0.362 μg) of the method are in accordance with high-quality requirements.

Following the same principles (small sample volume, high separation efficiency, and short analysis time), capillary electrophoresis (CE) was successfully used in berberine analysis. Du and Wang (2010) used CE with end-column electrochemiluminescence (ECL) detection for berberine analysis in both tablets and *Rhizoma coptidis*. Using a 4 min analysis time, a small sample volume (3.3 nL) and a LOD of (5 × 10^−9^ g/mL), the developed method proved to be highly sensitive and with good resolution (Du and Wang, 2010).

Besides UV, HPLC, HTPLC, TLC, and CE, other detection methods like liquid chromatography coupled with mass spectrometry (LC/MS) are currently employed to quantify berberine in biological fluids. Generally, it is considered a powerful technique for the analysis of complex samples because it offers rapid and accurate information about the structural composition of the compounds, especially when tandem mass spectrometry (MS^n^) is applied. For example, Xu et al. (2015) developed a sensitive an accurate LC-MS/MS method to determine berberine and other seven components in rat plasma using multiple reactions monitoring (MRM) mode. Compounds separation was optimized using six different types of reverse-phase columns, and two different mobile phases (methanol–water and acetonitrile–water with different additives). Additives like formic acid, acetic acid, and ammonium acetate were added in different concentrations as follows: 0.1, 0.5, 1, and 2% for formic acid, 0.1, 0.5, 1, and 2% for acetic acid and 0.0001, 0.001, 0.01 mol/L for ammonium acetate. The method was also tested in terms of specificity, linearity, lower limit of quantification (LLOQ), precision, accuracy, and stability (Xu et al., 2015).

## Antioxidant effect

Under normal conditions, the body maintains a balance between the antioxidant and pro-oxidant agents (reactive oxygen species—ROS and reactive nitrogen species—RNS; Rahal et al., 2014).

The imbalance between pro and antioxidants occurs in case of increased oxidative stress (Bhattacharyya et al., 2014).

The oxidative stress builds up through several mechanisms: an increase in the production of reactive species, a decrease in the levels of enzymes involved in blocking the actions of pro-oxidant compounds, and/or the decrease in free radical scavengers (Pilch et al., 2014).

An experimental study demonstrated the effect of berberine on lipid peroxidation after inducing chemical carcinogenesis in small animals (rats). An increase in LPO (lipid peroxidation) was observed after carcinogenesis induction, but also its significant reversal after berberine administration (30 mg/kg). Berberine shows therefore at least partial antioxidant properties, due to its effect on lipid peroxidation (Thirupurasundari et al., 2009).

Other mechanisms involved in the antioxidant role of berberine are: ROS/RNS scavenging, binding of metals leading to the transformation/oxidation of certain substances, free-oxygen removal, reducing the destructiveness of superoxide ions and nitric oxide, or increasing the antioxidant effect of some endogenous substances. The antioxidant effect of berberine was comparable with that of vitamin C, a highly-potent antioxidant (Shirwaikar et al., 2006; Ahmed et al., 2015).

The increase in blood sugar leads to oxidative stress not by generating oxygen reactive species but by impairing the antioxidant mechanisms. Administration of berberine to rats with diabetes mellitus increased the SOD (superoxide dismutase) activity and decreased the MDA (malondialdehyde) level (marker of lipid peroxidation). This antioxidant effect of berberine could explain the renal function improvement in diabetic nephropathy (Liu et al., 2008b).

The oxidative stress plays an important role in the pathogenesis of many diseases. The beneficial effect of berberine is presumed to reside mostly in its antioxidant role.

## Cardiovascular effects of berberine

### Effect on cardiac contractility

The beneficial effect of berberine in cardiac failure was demonstrated in a study on 51 patients diagnosed with NYHA (New York Heart Association) III/IV cardiac failure with low left ventricular ejection fraction (LVEF) and premature ventricular contractions and/or ventricular tachycardia. These patients received tablets containing 1.2 g berberine/day, together with conventional therapy (diuretics, ACEI—angiotensin-converting-enzyme inhibitors, digoxin, nitrates) for 2 weeks. An increase in LVEF was observed in all patients after this period, but also a decrease in the frequency and complexity of premature ventricular contractions. The magnitude of the beneficial effect was in direct proportion with the plasma concentration of berberine (Zeng, 1999).

### The cardioprotective effect during ischemia

Berberine can provide cardio-protection in ischemic conditions by playing various roles at different levels: modulation of AMPK (AMP—activated kinase) activity, AKT (protein kinase B) phosphorylation, modulation of the JAK/STAT (Janus kinase/signal transducers and activators of transcription) pathway and of GSK3β (glycogen synthase kinase 3β; Chang et al., 2016). AMPK is an important enzyme playing an essential role in cellular metabolism and offering protection in ischemic conditions by adjusting the carbohydrate and lipid metabolism, the function of cell organelles (mitochondria, endoplasmic reticulum) and the apoptosis (Zaha et al., 2016).

Berberine activates the PI3K (phosphoinositide 3-kinase)/AKT pathway which is considered a compensatory mechanism limiting the pro-inflammatory processes and apoptotic events in the presence of aggressive factors. The activation of this pathway is associated with a reduction of the ischemic injury through the modulation of the TLR4 (toll-like receptor 4)-mediated signal transduction (Hua et al., 2007).

Several supporting data indicate that the JAK2/STAT3 signaling plays an important role in cardioprotection against ischemia-reperfusion injury (Mascareno et al., 2001).

GSK3β is a serine/threonine protein-kinase, an enzyme involved in reactions associated to important processes at the cellular level: metabolization, differentiation, proliferation, and apoptosis. Berberine inhibits this kinase, thereby exercising its cardioprotective effect (Park et al., 2014).

## Effects on the endothelium

Berberine induces endothelial relaxation by increasing NO production from arginine through the activity of eNOS (endothelial nitric oxide synthase) which is considered a key element in the vasodilation process. Besides increasing the NO level, it also up-regulates eNOS mRNA. Furthermore, berberine facilitates the phosphorylation of eNOS and its coupling to HSP 90 (heat shock proteins), which consequently increases NO production (Wang et al., 2009).

Moreover, berberine reduces endothelial contraction by reducing COX-2 expression. Any imbalance in COX 1 or 2 activity may alter the ratio between prothrombotic/antithrombotic and vasodilator/vasoconstrictor effects (Liu L. et al., 2015).

The beneficial effect of berberine on the TNFα-induced endothelial contraction was also recorded, as well as an increase in the level of PI3K/AKT/eNOS mRNA (Xiao et al., 2014).

## The role of berberine in atherosclerosis

Atherogenesis is a consequence of high blood lipid levels and is associated with inflammatory changes in the vascular wall. Berberine interferes with this process by up-regulating the expression of SIRT1 (silent information regulator T1) and by inhibiting the expression of PPARγ (peroxisome proliferator-activated receptor-γ). SIRT1 is a NAD-dependent deacetylase. The SIRT1 enzyme has many targets (PPARγ, p53), all playing different roles in atherogenesis (Chi et al., 2014).

### The role of berberine in lipid metabolism

The effects of berberine on lipid metabolism are also the consequence of its effects on LDL cholesterol receptors. On one hand, these receptors are stabilized by an extracellular signal-regulated kinase (ERK)-dependent pathway, and on the other, berberine increases the activity of LDL receptors through the JNK pathway (Cicero and Ertek, 2009).

Moreover, berberine has an effect on ACAT (cholesterol acyltransferases), a class of enzymes that transform cholesterol into esters, thus playing an essential role in maintaining cholesterol homeostasis in different tissues. There are two types of ACAT enzymes, ACAT1, and ACAT2. ACAT1 is a ubiquitous enzyme, while ACAT2 can be found only in hepatic cells and enterocytes. Berberine influences the activity of ACAT2 without an effect on ACAT1, therefore reducing the intestinal absorption of cholesterol and decreasing its plasmatic level (Chang et al., 2009; Wang et al., 2014).

The hypolipidemic effect of berberine is also a result of its action on PCSK9 (proprotein convertase subtilisin kexin 9). This enzyme can attach itself to LDL receptors, leading to a decrease in LDL metabolization and an increase in its blood level (Xiao et al., 2012).

In a clinical trial, 63 patients with dyslipidemia were randomly divided in three groups. The first group was treated with berberine (1,000 mg/day), the second with simvastatin (20 mg/day) and the third with a combination of berberine and simvastatin. The authors reported a 23.8% reduction in LDL-C levels in patients treated with berberine, a 14.3% reduction in those treated with simvastatin and a 31.8% LDL-C reduction in the group treated with both simvastatin and berberine. This result demonstrates that berberine can be used alone or in association with simvastatin in the treatment of dyslipidemia (Kong et al., 2008).

## The role of berberine in glucose metabolism

Many studies demonstrated that berberine lowers blood sugar, through the following mechanisms:

- Inhibition of mitochondrial glucose oxidation and stimulation of glycolysis, and subsequently increased glucose metabolization (Yin et al., 2008a).
- Decreased ATP level through the inhibition of mitochondrial function in the liver, which may be the probable explanation of gluconeogenesis inhibition by berberine (Xia et al., 2011).
- Inhibition of DPP 4 (dipeptidyl peptidase-4), a ubiquitous serine protease responsible for cleaving certain peptides, such as the incretins GLP1 (glucagon-like peptide-1) and GIP (gastric inhibitory polypeptide); their role is to raise the insulin level in the context of hyperglycemia. The DPP4 inhibition will prolong the duration of action for these peptides, therefore improving overall glucose tolerance (Al-masri et al., 2009; Seino et al., 2010).

Berberine has a beneficial effect in improving insulin resistance and glucose utilization in tissues by lowering the lipid (especially triglyceride) and plasma free fatty acids levels (Chen et al., 2011).

The effect of berberine (1,500 mg day) on glucose metabolism was also demonstrated in a pilot study enrolling 84 patients with type 2 diabetes mellitus. The effect, including on HbA1c, was comparable to that of metformin (1,500 mg/day), one of the most widely used hypoglycemic drugs. In addition, berberine has a favorable influence on the lipid profile, unlike metformin, which has barely any effect (Yin et al., 2008b).

## Hepatoprotective effect of berberine

The hepatoprotective effect of berberine was demonstrated on lab animals (mice), in which hepatotoxicity was induced by doxorubicin. Pretreatment with berberine significantly reduced both functional hepatic tests and histological damage (inflammatory cellular infiltrate, hepatocyte necrosis; Zhao et al., 2012).

The mechanism by which berberine reduces hepatotoxicity was also studied on CCl_4_ (carbon tetrachloride)-induced hepatotoxicity. Berberine lowers the oxidative and nitrosamine stress and also modulates the inflammatory response in the liver, with favorable effects on the changes occurring in the liver. Berberine prevents the decrease in SOD activity and the increase in lipid peroxidation and contributes to the reduction in TNF-α, COX-2, and iNOS (inducible nitric oxide synthase) levels. The decrease in transaminase levels supports the hypothesis according to which berberine helps maintain the integrity of the hepatocellular membrane (Domitrović et al., 2011).

## Nephroprotective effect of berberine

The chronic kidney damage occurring in time in patients with HT (hypertension) and DM (diabetes mellitus) is well known; it is mainly due to the atherosclerosis of the renal artery, caused by inflammation and oxidative stress. The protective effect of berberine on kidneys was studied on 69 patients suffering from both HT and DM, with blood pressure and blood sugar levels controlled with conventional medication. The patients received 300 mg berberine/day for 24 months, with 2-week interruptions every 5 months. The authors recorded lower CRP (C-reactive protein), MDA and SOD levels after treatment, but without significant changes in creatinine, arterial pressure, or glycaemia levels. These results support the renal protective effect of berberine through its anti-inflammatory and antioxidant effects (Dai et al., 2015).

Another animal study tested the renoprotective effect of berberine after administration of HgCl_2_ (mercury chloride). This substance induces hepato-renal damage by increasing the oxidative stress (increases lipid peroxidation and NO levels, and lowers glutathione and SOD levels as well as the activity of other protective enzymes). Administration of HgCl_2_ increased the AST (aspartate aminotransferase), ALT (alanine aminotransferase), and ALP (alkaline phosphatase) levels, compared to the control group. However, pretreatment with berberine lowered these enzymes significantly. In addition, both urea and creatinine levels were significantly increased in the HgCl_2_ group vs. the control group, and again pretreatment with berberine prevented these changes. Additionally, the authors recorded higher pro-oxidant and lower antioxidant levels in the intervention group. These data support the hepatic and renal protective effects of berberine. Other studies performed on animal models with CCl_4−_induced hepatotoxicity demonstrated the same effect (Othman et al., 2014).

In addition, berberine can lower the nephrotoxicity caused by cisplatine. In an animal study, berberine was administered in progressive doses of 1, 2, 3 mg/kg, orally, for 2 successive days, starting 2 days after cisplatine administration. After the last doses of berberine, the animals were sacrificed and the kidneys were examined by the pathologist. The results showed significant histological improvement and a reduction in NF-kB (nuclear factor kappa-light-chain-enhancer of activated B cells), TNF α, COX2 an iNOS levels, all of which support the anti-inflammatory effect of berberine (Domitrović et al., 2013).

## Immunomodulatory effect of berberine

The immunomodulatory effect of berberine was demonstrated in many experimental and clinical contexts.

In an experimental autoimmune myocarditis model, berberine contributed to mitigate the cardiac damage by: limiting the rise in anticardiac myosin antibodies, modulating the activity of certain STATs and blocking Th1 and Th2 cell differentiation, which play an important role in the pathogenesis of myocarditis (Liu X. et al., 2016).

Experimental autoimmune neuritis is an experimental animal model equivalent to the Guillain-Barre syndrome in humans. This neurologic syndrome is characterized by autoimmune injury of the peripheral nervous system. The beneficial effect of berberine on this animal model resided in its influence on cellular and humoral immunity through the inhibition of lymphocyte proliferation (especially CD4), and the decrease in pro-inflammatory cytokines (IL-6 and TNF α; Li et al., 2014).

Experimental autoimmune encephalomyelitis is an established model of multiple sclerosis. Multiple sclerosis is a one of the most common diseases of the central nervous system (CNS) and involves neurodegenerative and inflammatory processes, and autoimmune demyelination (Ransohoff et al., 2015). The blood-brain barrier permeability and changes in matrix metalloproteinase (MMP) levels in the cerebrospinal fluid and brain were studied using this model (Ma et al., 2010). MMPs may be involved in demyelination and their activity in tissues depends on the balance between their level and their tissue inhibitors. MMP2 and MMP9 are the main endoproteinases involved in the migration of lymphocytes in CNS and in altering the BBB (blood brain barrier) (Avolio et al., 2003). Berberine has a beneficial effect in experimental autoimmune encephalomyelitis by inhibiting the activity of MMP9, reducing BBB permeability and, consecutively, by decreasing the inflammatory cellular infiltration of the CNS (Ma et al., 2010).

**Figure 3:**
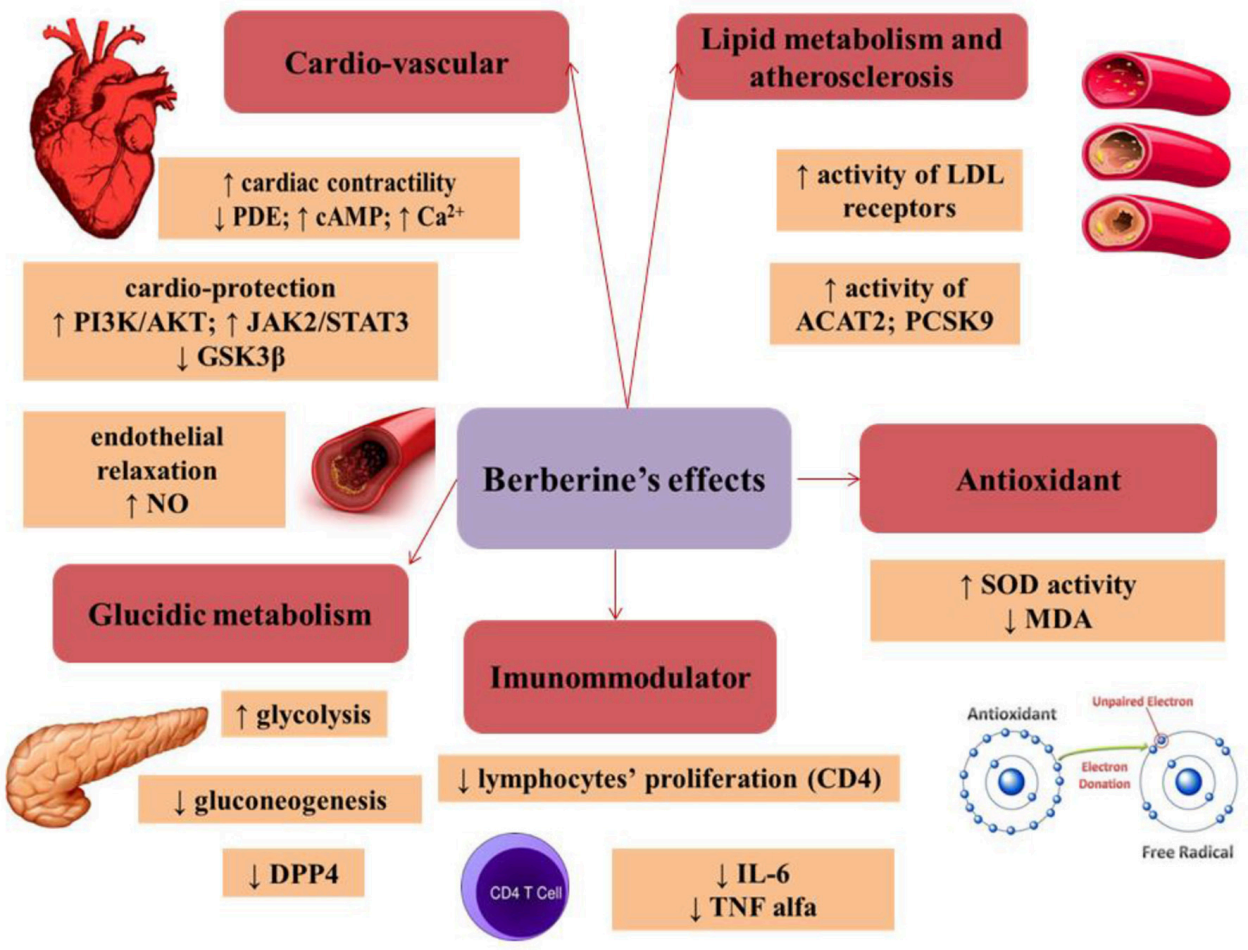
Therapeutic effects of berberine *in vivo*. Mechanisms of berberine in regulation of metabolism, immunity and oxidative reactions. Phosphodiesterase (PDE), cyclic 3′,5′-adenosine monophosphate (cAMP), phosphoinositide 3-kinase/protein kinase B (PI3K/AKT), Janus kinase/signal transducers and activators of transcription (JAK/STAT), glycogen synthase kinase 3β (GSK3β), superoxide dismutase (SOD), malondialdehyde (MDA), nitric oxide (NO), cholesterol acyltransferases (ACAT2), dipeptidyl peptidase-4 (DPP 4), proprotein convertase subtilisin kexin 9 (PCSK9).

The current therapy used for inflammatory bowel diseases, including glucocorticoids and immunosuppressive agents, has a low level of safety. The effect of berberine was studied in combination with 5-ASA (5-aminosalicylic acid) vs. 5-ASA alone using an experimental animal model with DSS (dextran sulfate sodium)-induced colitis. The authors analyzed the level of proinflammatory cytokines in the animal gut. A decrease in COX2, IL6, and IL23 mRNA levels was observed in animals treated only with 5-ASA, whereas animals treated with both 5-ASA and berberine had a reduction in mRNA levels for COX2, IL6, IL23 as well as for TNF alfa and IL12b. This beneficial effect could partially be attributed to the inhibition of NF-kB and the reduction in JAK2 phosphorylation (through the influence on the JAK/STAT pathway) by both 5-ASA and berberine (Li et al., 2015; Figure 3).

Another study demonstrated that berberine increases the corticosteroid level in rats with experimentally-induced colitis. This engendered the theory that its beneficial effect may also be attributed to the increase in endogenous glucocorticoid levels, compounds with well-known therapeutic effect in inflammatory bowel disease (Minaiyan et al., 2011).

## Conclusion

A review of the available scientific literature shows that the traditional medical uses of berberine-containing plants have been evaluated by modern pharmacological studies. Different species of berberine-rich plants have multiple pharmacological and therapeutic actions, such as antioxidant and immunomodulatory effects, protective action on the cardiovascular system, liver and kidney, endothelial relaxation, regulator on glucose metabolism and atherosclerosis, which can all be explained by the presence of berberine as well as other phyto constituents (when dealing with berberine-containing plant extracts). Moreover, the effects of berberine vary according to its origin (different plants or pharmaceutical products) and its concentration, depending on the very diverse extraction and detection techniques already described. Over time, modern extraction techniques were increasingly preferred to classical ones. Since classical methods are generally time- and solvent-consuming processes, modern extraction techniques such as USE, MAE, UPE, SFE, and PLE are seen as better alternatives to overcome these limitations. Furthermore, berberine, due to its antioxidant and anti-inflammatory effects, has several clinical applications in many disorders, from inflammatory conditions to the metabolic syndrome. However, there are some traditional uses that have not yet been completely elucidated, and further studies are needed. Therefore, extensive studies on the potential of plants containing berberine that have shown aforementioned pharmacological activities should go through additional *in vitro* and *in vivo* studies.

## Author contributions

MN, AM, JE, and RP have conceived and designed the structure of the manuscript, data collection, and drafting, as well as its revision. CB, GC, and AB have critically reviewed the manuscript. All authors have seen and agreed on the final version of the manuscript.

### Conflict of interest statement

The authors declare that the research was conducted in the absence of any commercial or financial relationships that could be construed as a potential conflict of interest.

## References

[B1] Abbasi A. M. Dastagir G. Hussain F. Sanaullah P. (2005). Ethnobotany and marketing of crude drug plants in district Haripur, Pakistan. Pak. J. Plant Sci. 11, 103–114.

[B2] Abbasi A. M. Khan M. A. Ahmad M. Zafar M. Jahan S. Sultana S. (2010). Ethnopharmacological application of medicinal plants to cure skin diseases and in folk cosmetics among the tribal communities of North-West Frontier Province, Pakistan. J. Ethnopharmacol. 128, 322–335. 10.1016/j.jep.2010.01.052 20138210

[B3] Abbasi A. M. Khan M. A. Ahmad M. Zafar M. Khan H. Muhammad N. (2009). Medicinal plants used for the treatment of jaundice and hepatitis based on socio-economic documentation. *Afr. J*. Biotechnol. 8, 1643–1650.

[B4] Abou-Donia A. H. A. El-Din A. A. S. (1986). Phytochemical study of *Argemone mexicana* L. grown in Egypt. *Egypt. J. Pharm*. Sci. 25, 1–5.

[B5] Acharya K. P. Rokaya M. B. (2005). Ethnobotanical survey of medicinal plants traded in the streets of Kathmandu valley. *Sci*. World 3, 44–48.

[B6] Adeyemi A. A. Gbolade A. A. Moody J. O. Ogbole O. O. Fasanya M. T. (2010). Traditional anti-fever phytotherapies in Sagamu and Remo north districts in Ogun State, Nigeria. J. Herbs. Spices Med. Plants 16, 203–218. 10.1080/10496475.2010.511075

[B7] Adjanohoun J. E. Aboobakar N. Dramane K. (1996). Traditional Medicine and Pharmacopoeia: Contribution to Ethnobotanical and Floristic Studies in Cameroon. Porto-Novo: Technical and Research Commission (STRC) of the Organization of African Unity.

[B8] Ahmed E. Arshad M. Ahmad M. Saeed M. Ishaque M. (2004). Ethnopharmacological survey of some medicinally important plants of Galliyat Areas of NWFP, Pakistan. Asian J. Plant Sci. 3, 410–415. 10.3923/ajps.2004.410.415

[B9] Ahmed T. Gilani A. U. Abdollahi M. Daglia M. Nabavi S. F. Nabavi S. M. (2015). Berberine and neurodegeneration: a review of literature. Pharmacol. Rep. 67, 970–979. 10.1016/j.pharep.2015.03.002 26398393

[B10] Ahn D. K. (2003). Illustrated Book of Korean Medicinal Herbs. Seoul: Kyo-Hak Publishing, Kyohaksa.

[B11] Ajali U. (2000). Antibacterial activity of *Enantia polycarpa* bark. Fitoterapia 71, 315–316. 10.1016/S0367-326X(99)00153-7 10844170

[B12] Akowuah G. A. Okechukwu P. N. Chiam N. C. (2014). Evaluation of HPLC and spectrophotometric methods for analysis of bioactive constituent berberine in stem extracts of *Coscinium fenestratum*. Acta Chromatogr. 26, 243–254. 10.1556/AChrom.26.2014.2.4

[B13] Al-Douri N. A. (2000). A survey of medicinal plants and their traditional uses in Iraq. Pharm. Biol. 38, 74–79. 10.1076/1388-0209(200001)3811-BFT074 21214444

[B14] Ali M. Shah S. Z. Khan M. S. Naz M. F. R. Zafar A. (2018). Ethnobotanical study on the weeds of wheat crop in district Swabi, Khyber Pakhtunkhwa, Pakistan. Int. J. Biosci. 12, 363–374. 10.12692/ijb/12.1.363-374

[B15] Al-masri I. M. Mohammad M. K. Tahaa M. O. (2009). Inhibition of dipeptidyl peptidase IV (DPP IV) is one of the mechanisms explaining the hypoglycemic effect of berberine. J. Enzyme Inhib. Med. Chem. 24, 1061–1066. 10.1080/14756360802610761 19640223

[B16] Al-Qura'n S. (2009). Ethnopharmacological survey of wild medicinal plants in Showbak, Jordan. J. Ethnopharmacol. 123, 45–50. 10.1016/j.jep.2009.02.031 19429338

[B17] Alupului A. Calinescu I. Lavric V. (2009). Ultrasonic vs. microwave extraction intensification of active principles from medicinal plants. Chem. Eng. Trans. 17, 1023–1028. 10.3303/cet0917171

[B18] Andola H. C. Gaira K. S. Rawal R. S. Rawat M. S. Bhatt I. D. (2010a). Habitat-dependent variations in berberine content of *Berberis asiatica* Roxb. ex. DC. in Kumaon, Western Himalaya. Chem. Biodivers. 7, 415–420. 10.1002/cbdv.200900041 20151388

[B19] Andola H. C. Rawal R. S. Rawat M. S. M. Bhatt I. D. Purohit V. K. (2010b). Variations of berberine contents in *Berberis pseudumbellata*: a high value medicinal shrub of west Himalaya, India. Med. Plants Int. J. Phytomed. Relat. Ind. 2, 111–115. 10.5958/j.0975-4261.2.2.017

[B20] Andola H. C. Rawal R. S. Rawat M. S. M. Bhatt I. D. Purohit V. K. (2010c). Analysis of berberine content using HPTLC fingerprinting of root and bark of three Himalayan *Berberis* species. Asian J. Biotechnol. 2, 239–245. 10.3923/ajbkr.2010.239.245

[B21] Anesini C. Perez C. (1993). Screening of plants used in Argentine folk medicine for antimicrobial activity. J. Ethnopharmacol. 39, 119–128. 10.1016/0378-8741(93)90027-3 8412245

[B22] Arawwawala L. D. A. M. Wickramaar W. A. N. (2012). Berberine content in *Coscinium fenestratum* (Gaertn.) Colebr grown in Sri Lanka. Pharmacologia 3, 679–682. 10.5567/pharmacologia.2012.679.682

[B23] Arayne M. S. Sultana N. Bahadur S. S. (2007). The berberis story: *Berberis vulgaris* in therapeutics. *Pak. J. Pharm*. Sci. 20, 83–92. 17337435

[B24] Atta-ur-Rahma Ahmad H. (1992). An aporphine-benzylisoquinoline alkaloid from *Berberis waziristanica*. Phytochemistry 31, 1835–1836. 10.1016/0031-9422(92)83163-S

[B25] Avolio C. Ruggieri M. Giuliani F. Liuzzi G. M. Leante R. Riccio P.. (2003). Serum MMP-2 and MMP-9 are elevated in different multiple sclerosis subtypes. J. Neuroimmunol. 136, 46–53. 10.1016/S0165-5728(03)00006-7 12620642

[B26] Babu N. H. R. Thriveni H. N. Vasudeva R. (2012). Influence of drying methods and extraction procedures on the recovery of berberine content in *Coscinium fenestratum. J. Nat. Prod*. Plant Resour. 2, 540–544.

[B27] Bahar M. Deng Y. Zhu X. He S. Pandharkar T. Drew M. E.. (2011). Potent antiprotozoal activity of a novel semi-synthetic berberine derivative. Bioorg. Med. Chem. Lett. 21, 2606–2610. 10.1016/j.bmcl.2011.01.101 21474310

[B28] Baharvand-Ahmadi B. Bahmani M. Tajeddini P. Naghdi N. Rafieian-Kopaei M. (2016). An ethno-medicinal study of medicinal plants used for the treatment of diabetes. J. Nephropathol. 5, 44–50. 10.15171/jnp.2016.08 27047810 PMC4790187

[B29] Baldazzi C. Leone M. G. Casini M. L. Tita B. (1998). Effects of the major alkaloid of *Hydrastis canadensis* L., berberine, on rabbit prostate strips. Phyther. Res. 12, 589–591. 10.1002/(SICI)1099-1573(199812)12:8<589::AID-PTR347>3.0.CO;2-I

[B30] Bapna S. Choudhary P. K. Ramaiya M. Chowdhary A. (2015). Antiplasmodial activity of *Argemone mexicana*: an *in vivo* and *in vitro* study. *World J. Pharm*. Res. 4, 1653–1663.

[B31] Bele M. Y. Focho D. A. Egbe E. A. Chuyong B. G. (2011). Ethnobotanical survey of the uses Annonaceae around mount Cameroon. Afr. J. Plant Sci. 5, 237–247.

[B32] Betti J. L. Caspa R. Ambara J. Kourogue R. L. (2013). Ethno-botanical study of plants used for treating malaria in a forest: savanna margin area, East region, Cameroon. *Glob. J. Res. Med. Plants Indig*. Med. 2, 692.

[B33] Betti J. L. Lejoly J. (2009). Contribution to the knowledge of medicinal plants of the Dja Biosphere Reserve, Cameroon: plants used for treating jaundice. J. Med. Plants Res. 3, 1056–1065.

[B34] Bhandari D. K. Nath G. Ray A. B. Tewari P. V. (2000). Antimicrobial activity of crude extracts from *Berberis asiatica* stem bark. Pharm. Biol. 38, 254–257. 10.1076/1388-0209(200009)3841-AFT254 21214470

[B35] Bhattacharjee S. Tiwari K. C. Majumdar R. Misra A. K. (1980). Folklore medicine from district Kamrup (Assam). Bull. Medic. Ethno. Bot. Res. 1, 447–460.

[B36] Bhattacharyya A. Chattopadhyay R. Mitra S. Crowe S. E. (2014). Oxidative stress: an essential factor in the pathogenesis of gastrointestinal mucosal diseases. Physiol. Rev. 94, 329–354. 10.1152/physrev.00040.2012 24692350 PMC4044300

[B37] Birdsall T. C. (1997). Berberine: therapeutic potential of an alkaloid found in several medicinal plants. *Altern. Med*. Rev. 2, 94–103.

[B38] Bonesi M. Loizzo M. R. Conforti F. Passalacqua N. G. Saab A. Menichini F.. (2013). *Berberis aetnensis* and B. *libanotica:* A comparative study on the chemical composition, inhibitory effect on key enzymes linked to Alzheimer's disease and antioxidant activity. J. Pharm. Pharmacol. 65, 1726–1735. 10.1111/jphp.12172 24236982

[B39] Borokini T. I. Clement M. Dickson N. J. Edagbo D. E. (2013). Ethnobiological survey of traditional medicine practice for fevers and headaches in Oyo State, Nigeria. *Topclass J. Herb*. Med. 2, 121–130.

[B40] Bose B. C. Vijayvargiya R. Saifi A. Q. Sharma S. K. (1963). Chemical and pharmacological studies on *Argemone mexicana*. J. Pharm. Sci. 52, 1172–1175. 10.1002/jps.2600521216 14088969

[B41] Bouquet A. (1969). Féticheurs et Médecines Traditionnelles du Congo (Brazzaville). Mém. O.R.S.T.O.M. (Paris: Office la Rech. Sci. Tech. outre-mer) 36, 282.

[B42] Bouquet A. Debray M. (1974). Plantes Médicinales de la Côte d'Ivoire. Paris Off. la Rech. Sci. Tech. Paris: Outre Mer 231p. (Travaux Doc. l'ORSTOM no. 32) Illus., col. illus. Geog 5.

[B43] Bown D. (1995). Encyclopaedia of Herbs and Their Uses. London: Dorling Kindersley London.

[B44] Burkill H. M. (1985). The Useful Plants of West Tropical Africa. London: Royal Botanic Gardens, Kew.

[B45] Bushra I. Kishwar S. Qureshi R. A. Saddiqa M. (2000). A checklist of plants of Bhogarmang, Siran Valley NWFP, Pakistan. Hamdard Med. 43, 62–76.

[B46] Buzas A. Egnell C. (1965). On the presence of quinidine in addition to berberine alkaloids in the barks of *Enantia pilosa* and *Enantia polycarpa* (Annonaceae). *Ann. Pharm*. Fr. 23, 351. 5840950

[B47] Castleman M. (1991). The Healing Herbs: The Ultimate Guide to the Curative Powers of Nature's Medicine. Emmaus: Rodale Press.

[B48] Chakravarti K. K. Dhar D. C. Siddiqui S. (1950). Alkaloidal constituents of the bark of *Berberis aristata*. J. Sci. Ind. Res. 9, 161–164.

[B49] Chan C.-O. Chu C.-C. Mok D. K. Chau F.-T. (2007). Analysis of berberine and total alkaloid content in *Cortex phellodendri* by near infrared spectroscopy (NIRS) compared with high-performance liquid chromatography coupled with ultra-visible spectrometric detection. Anal. Chim. Acta 592, 121–131. 10.1016/j.aca.2007.04.016 17512816

[B50] Chandra P. Purohit A. N. (1980). Berberine contents and alkaloid profile of *Berberis* species from different altitudes. Biochem. Syst. Ecol. 8, 379–380. 10.1016/0305-1978(80)90040-X

[B51] Chang T.-Y. Li B.-L. Chang C. C. Urano Y. (2009). Acyl-coenzyme A:cholesterol acyltransferases. Am. J. Physiol. Endocrinol. Metab. 297, E1–E9. 10.1152/ajpendo.90926.2008 19141679 PMC2711667

[B52] Chang W. Li K. Guan F. Yao F. Yu Y. Zhang M.. (2016). Berberine pretreatment confers cardioprotection against ischemia-reperfusion injury in a rat model of type 2 diabetes. J. Cardiovasc. Pharmacol. Ther. 21, 486–494. 10.1177/1074248415627873 26846272

[B53] Chang Y. (2013). Ultrasonic-assisted extraction of berberine in ionic liquid. *Pharm*. Eng. 33, 1–4.

[B54] Chatterjee D. R. (1951). Plant alkaloids. I. *Berberis floribunda*. J. Indian Chem. Soc. 28, 225–228.

[B55] Chatterjee R. Banerjee A. (1953). Plant alkaloids. V. *Berberis lambertii*. J. Indian Chem. Soc. 30, 705–707.

[B56] Chatterjee R. Guha M. P. Das Gupta A. K. (1952). Plant alkaloids. IV. *Berberis himalaica* and *B. tinctoria*. J. Indian Chem. Soc. 29, 921–924.

[B57] Chaudhury R. H. N. Guha A. Chaudhury R. Pal D. C. (1980). Ethnobotanical uses of herbaria-2. *J. Econ. Taxon*. Bot. 1, 163–168.

[B58] Chen A. H. (1981). Studies on the analysis of alkaloids of *Phellodendron wilsonii* Hay. et Kaneh. Kaneh. Kexue Fazhan Yuekan 9, 398–411.

[B59] Chen A. H. (1982). Applied studies on the alkaloids of *Phellodendron wilsonii* Hay. et Kaneh. II. the alkaloid contents in Taiwan plants. Kexue Fazhan Yuekan 10, 279–286.

[B60] Chen C. Yu Z. Li Y. Fichna J. Storr M. (2014). Effects of berberine in the gastrointestinal tract — a review of actions and therapeutic implications. Am. J. Chin. Med. 42, 1053–1070. 10.1142/S0192415X14500669 25183302

[B61] Chen H. F. Chen C. M. (1988). Determination of berberine in crude and processed Chinese herb: *Coptidis rhizoma* and *Phellodendri cortex*. Zhonghua Yaoxue Zazhi 40, 259–264.

[B62] Chen W. H. Pang J. Y. Qin Y. Peng Q. Cai Z. Jiang Z. H. (2005). Synthesis of linked berberine dimers and their remarkably enhanced DNA-binding affinities. Bioorg. Med. Chem. Lett. 15, 2689–2692. 10.1016/j.bmcl.2004.10.098 15863343

[B63] Chen Y. Wang Y. Zhang J. Sun C. Lopez A. (2011). Berberine improves glucose homeostasis in streptozotocin-induced diabetic rats in association with multiple factors of insulin resistance. ISRN Endocrinol. 2011, 1–8. 10.5402/2011/519371 22363882 PMC3262646

[B64] Chen Y. Y. Chang F. R. Wu Y. C. (1996). Isoquinoline alkaloids and lignans from *Rollinia mucosa*. J. Nat. Prod. 59, 904–906. 10.1021/np960414z

[B65] Chevallier A. (1996). The Encyclopedia of Medicinal Plants. London: Dorling Kindersley.

[B66] Chhetri D. R. Parajuli P. Subba G. C. (2005). Antidiabetic plants used by Sikkim and Darjeeling Himalayan tribes, India. J. Ethnopharmacol. 99, 199–202. 10.1016/j.jep.2005.01.058 15894127

[B67] Chi L. Peng L. Pan N. Hu X. Zhang Y. (2014). The anti-atherogenic effects of berberine on foam cell formation are mediated through the upregulation of sirtuin 1. Int. J. Mol. Med. 34, 1087–1093. 10.3892/ijmm.2014.1868 25069720

[B68] Chiang Y. L. Su C. R. Kuo P. C. Damu A. G. Wu T. S. (2006). Two isoquinolones from the roots of *Phellodendron amurense* var. Wilsonii. Heterocycles 68, 339–345. 10.3987/COM-05-10598

[B69] Chopra R. N. Nayar S. I. Chopra I. C. (1986). Glossary of Indian Medicinal Plants (Including the Supplement). New Delhi: Canal of Scientific and Industrial Research.

[B70] Cicero A. Ertek S. (2009). Berberine: metabolic and cardiovascular effects in preclinical and clinical trials. Nutr. Diet Suppl. 1, 1–10. 10.2147/NDS.S6084

[B71] Coffey T. (1993). The History and Folklore of North American Wildflowers. New York, NY: Facts on File Limited.

[B72] Dai P. Wang J. Lin L. Zhang Y. Wang Z. (2015). Renoprotective effects of berberine as adjuvant therapy for hypertensive patients with type 2 diabetes mellitus: evaluation via biochemical markers and color Doppler ultrasonography. Exp. Ther. Med. 10, 869–876. 10.3892/etm.2015.2585 26622407 PMC4533140

[B73] de Almeida Costa O. (1935). (Mexican poppy) *Argemone mexicana L. Rev. Flora Med* 1, 271–282.

[B74] Deng A. J. Qin H. L. (2010). Cytotoxic dihydrobenzophenanthridine alkaloids from the roots of *Macleaya microcarpa*. Phytochemistry 71, 816–822. 10.1016/j.phytochem.2010.02.007 20226485

[B75] Deng Y. Liao Q. Li S. Bi K. Pan B. Xie Z. (2008). Simultaneous determination of berberine, palmatine and jatrorrhizine by liquid chromatography-tandem mass spectrometry in rat plasma and its application in a pharmacokinetic study after oral administration of coptis-evodia herb couple. J. Chromatogr. B Anal. Technol. Biomed. Life Sci. 863, 195–205. 10.1016/j.jchromb.2007.12.028 18258496

[B76] Dev S. (2006). A Selection of Prime Ayurvedic Plants Drugsancient- Modern Concordance. New Delhi: Anamaya Publishers.

[B77] Din N. Dibong S. D. Mpondo E. M. Priso R. J. Kwin N. F. Ngoye A. (2011). Inventory and identification of plants used in the treatment of diabetes in douala town (Cameroon). Eur. J. Med. Plants 1, 60–73. 10.9734/EJMP/2011/273

[B78] Doepke W. Ulrich H. Jimenez V. (1976). On the structure of a new alkaloid from *Argemone mexicana*. Z. Chem. 16, 54–55.

[B79] Domitrović R. Cvijanović O. Pernjak-Pugel E. Škoda M. Mikelić L. Crnčević-Orlić Ž. (2013). Berberine exerts nephroprotective effect against cisplatin-induced kidney damage through inhibition of oxidative/nitrosative stress, inflammation, autophagy and apoptosis. Food Chem. Toxicol. 62, 397–406. 10.1016/j.fct.2013.09.003 24025684

[B80] Domitrović R. Jakovac H. Blagojević G. (2011). Hepatoprotective activity of berberine is mediated by inhibition of TNF-α, COX-2, and iNOS expression in CCl4-intoxicated mice. Toxicology 280, 33–43. 10.1016/j.tox.2010.11.005 21095217

[B81] Doncheva T. Kostova N. Yordanova G. Saadi H. Akrib F. Dimitrov D. (2014). Comparison of alkaloid profile from *Glaucium corniculatum* (Papaveraceae) of Algerian and Bulgarian origin. Biochem. Syst. Ecol. 56, 278–280. 10.1016/j.bse.2014.07.007

[B82] Du J. X. Wang M. (2010). Capillary electrophoresis determination of berberine in pharmaceuticals with end-column electrochemiluminescence detection. J. Chinese Chem. Soc. 57, 696–700. 10.1002/jccs.201000097

[B83] Duke J. A. Ayensu E. S. (1985). Medicinal Plants of China. Algonac, MI: Reference Publications.

[B84] Duke J. A. Beckstrom-Sternberg S. M. (1994). Dr. Duke's phytochemical and ethnobotanical databases. Available online at: http://www.ars-grin.gov/duke/plants.html (Accessed January 15, 2017).

[B85] Dzhalilov D. R. Goryaev M. I. Kruglykhina G. K. (1963). Alkaloids from *Berberis iliensis*. I Izv. Akad. Nauk Kaz. SSR, Ser. Tekhn. i Khim. Nauk 3, 15–19.

[B86] Egels W. (1959). *Papaver dubium* var. *lecoquii*, a berberine-containing poppy. Planta Med. 7, 92–102. 10.1055/s-0028-1101592

[B87] Ehiagbonare P. O. Onyibe J. (2008). Conservation studies on four medicinal taxa of Southern Nigeria. *Sci. Res*. Essays 3, 40–45.

[B88] El Beyrouthy M. Arnold N. Delelis-Dusollier A. Dupont F. (2008). Plants used as remedies antirheumatic and antineuralgic in the traditional medicine of Lebanon. J. Ethnopharmacol. 120, 315–334. 10.1016/j.jep.2008.08.024 18809483

[B89] Emboden W. (1979). Narcotic Plants. New York, NY: Collier.

[B90] Emes M. Aguilar A. Argueta A. Cano L. (1994). Indigenous Medicinal Florae from México, Vol. II.

[B91] Eric Brussell D. (2004). A medicinal plant collection from Montserrat, West Indies. Econ. Bot. 58, S203–S220. 10.1663/0013-0001(2004)58[S203:AMPCFM]2.0.CO;2

[B92] Esseily F. El Ezzy M. Gali-Muhtasib H. Safi S. Esseily J. Diab-Assaf M. (2012). The ethanol fraction from the stem of *Berberis libanotica* inhibits the viability of adult T cell leukemia. Minerva Biotecnol. 24, 129–133.

[B93] Etminan M. Gill S. S. Samii A. (2005). Intake of vitamin E, vitamin C, and carotenoids and the risk of Parkinson's disease: a meta-analysis. Lancet. Neurol. 4, 362–365. 10.1016/S1474-4422(05)70097-1 15907740

[B94] Fabricant D. S. Farnsworth N. R. (2001). The value of plants used in traditional medicine for drug discovery. Environ. Heal. Perspect. Suppl. 109:69. 10.1289/ehp.01109s169 11250806 PMC1240543

[B95] Farías-Campomanes A. M. Rostagno M. A. Coaquira-Quispe J. J. Meireles M. A. A. (2015). Supercritical fluid extraction of polyphenols from lees: overall extraction curve, kinetic data and composition of the extracts. Bioresour. Bioprocess. 2, 45 10.1186/s40643-015-0073-5

[B96] Feng J. Xu W. Tao X. Wei H. Cai F. Jiang B.. (2010). Simultaneous determination of baicalin, baicalein, wogonin, berberine, palmatine and jatrorrhizine in rat plasma by liquid chromatography-tandem mass spectrometry and application in pharmacokinetic studies after oral administration of traditional Chinese medicinal preparations containing *Scutellaria-Coptis* herb couple. J. Pharm. Biomed. Anal. 53, 591–598. 10.1016/j.jpba.2010.04.002 20430560

[B97] Fletcher M. T. Takken G. Blaney B. J. Alberts V. (1993). Isoquinoline alkaloids and keto-fatty acids of *Argemone ochroleuca* and *A. mexicana* (Mexican poppy) seed. I. An assay method and factors affecting their concentration. Aust. J. Agric. Res. 44, 265–275. 10.1071/AR9930265

[B98] Fogarty J. E. (1990). A Barefoot Doctor's Manual: The American Translation of the Official Chinese Paramedical Manual. Philadelphia, PA: Running Press Book Publishers.

[B99] Fongod A. G. (2014). Ethnobotany, indigenous knowledge and unconscious preservation of the environment: An evaluation of indigenous knowledge in South and Southwest Regions of Cameroon. Int. J. Biodivers. Conserv. 6, 85–99. 10.5897/IJBC2013.0637

[B100] Foote P. A. (1932). The alkaloids of *Argemone* alba Lestib. J. Am. Pharm. Assoc. 21, 246–248.

[B101] Foster S. Duke J. A. (1990). A Field Guide to Medicinal Plants: Eastern and Central North America. Boston, MA: Houghton Mifflin Company.

[B102] Freile M. Giannini F. Sortino M. Zamora M. Juarez A. Zacchino S. (2006). Antifungal activity of aqueous extracts and of berberine isolated from *Berberis heterophylla. Acta Farm*. Bonaer. 25, 83–88.

[B103] Fyson P. F. (1975). Flora of the Nilgiri and Pulney Hill-Tops. Dehra Dun: Bishen Singh Mahendra Pal Singh and Periodical Experts.

[B104] Gbile Z. O. Soladoye M. O. Adesina S. K. (1988). Plants in traditional medicine in West Africa. Monogr. Syst. Bot. Missouri Bot. Gard. 25, 343–349.

[B105] Gbolade A. (2012). Ethnobotanical study of plants used in treating hypertension in Edo State of Nigeria. J. Ethnopharmacol. 144, 1–10. 10.1016/j.jep.2012.07.018 22975417

[B106] Gertig H. (1964). Alkaloids of *Eschscholtzia californica*. I. Isolation and thin-layer chromatography of alkaloid fractions from roots. Acta Pol. Pharm. 21, 59–64. 14193508

[B107] Gill L. S. Akinwumi C. (1986). Nigerian folk medicine: practices and beliefs of the Ondo people. J. Ethnopharmacol. 18, 257–266. 10.1016/0378-8741(86)90004-8 3821140

[B108] Gorval' L. M. Grishkovets V. I. (1999). Alkaloids of some species of the genus *Berberis* introduced into the Crimea. Chem. Nat. Compd. 35, 223–224. 10.1007/BF02234944

[B109] Govindasamy R. Simon J. Puduri V. S. Juliani H. R. Asante-Dartey J. Arthur H. (2007). Retailers and Wholesalers of African Herbal and Natural Products: Case Studies from Ghana and Rwanda. Issues New Crop. New Uses. Virginia ASHP, 332–337.

[B110] Greathouse G. A. (1939). Alkaloids from *Sanguinaria canadensis* and their influence on growth of *Phymatotrichum omnivorum*. Plant Physiol. 14, 377. 10.1104/pp.14.2.377 16653565 PMC437745

[B111] Grieve A. (1984). A Modern Herbal Penguin. Harmondsworth: Dover Publications Inc.

[B112] Grycová L. Dostál J. Marek R. (2007). Quaternary protoberberine alkaloids. Phytochemistry 68, 150–175. 10.1016/j.phytochem.2006.10.004 17109902

[B113] Gu Y. Zhang Y. Shi X. Li X. Hong J. Chen J.. (2010). Effect of traditional Chinese medicine berberine on type 2 diabetes based on comprehensive metabonomics. Talanta 81, 766–772. 10.1016/j.talanta.2010.01.015 20298851

[B114] Guoping L. Jinhong L. Shuai H. Jian C. Zhongyi Z. (2012). Optimization for ultrahigh pressure extraction of berberine from *Cortex phellodendri* by central composite design-response surface methodology. J. Med. Plants Res. 6, 3963–3970. 10.5897/JMPR11.1092

[B115] Gupta A. K. Tandon N. (2004). Rev. Indian Med. Plants, Vol 4. Delhi: ICMR.

[B116] Gurguel L. de Costa O. A. da Silva R. D. (1934). *Berberis laurina*. Anatomic, histologic and chemical study. Bol. Assoc. Bras. pharm. 15, 11–20.

[B117] Habtemariam S. (2011). The therapeutic potential of *Berberis darwinii* stem-bark: quantification of berberine and *in vitro* evidence for Alzheimer's disease therapy. Nat. Prod. Commun. 6, 1089–1090. 21922905

[B118] Haisova K. Slavik J. (1975). On the minor alkaloids from *Argemone mexicana* L. Collect Czech. Chem. Commun. 40, 1576–1578. 10.1135/cccc19751576

[B119] Hakim S. A. Mijovic V. Walker J. (1961). Distribution of certain poppy-fumaria alkaloids and a possible link with the incidence of glaucoma. Nature 189, 198–201. 10.1038/189198a0 13710637

[B120] Hamayun M. Khan A. Khan M. A. (2003). Common medicinal folk recipes of District Buner, NWFP, Pakistan. Ethnobot. Leafl. 2003, 14.

[B121] Hamonniere M. Leboeuf A. Paris R. R. (1975). Alcaloïdes des annonacées: alcaloïdes de l'*Enantia chlorantha*. Plant. Med. Phytother. 9, 296–303.

[B122] Hartwell J. L. (1982). Plants Used Against Cancer. Lawrence, MA: Quarterman Publications. Inc.

[B123] Hashmi K. Hafiz A. (1986). *In vivo* antibacterial activity of *Berberis asiatica*. J. Pak. Med. Assoc. 36, 5. 3084817

[B124] Hayta S. Polat R. Selvi S. (2014). Traditional uses of medicinal plants in Elazig (Turkey). J. Ethnopharmacol. 154, 613–623. 10.1016/j.jep.2014.04.026 24793217

[B125] He J.-M. Mu Q. (2015). The medicinal uses of the genus *Mahonia* in traditional Chinese medicine: an ethnopharmacological, phytochemical and pharmacological review. J. Ethnopharmacol. 175, 668–683. 10.1016/j.jep.2015.09.013 26387740

[B126] Henry T. A. (1949). The Plant Alkaloids, 4th Edn. Philadelphia, PA: Blakiston.

[B127] Hirschhorn H. H. (1981). Botanical remedies of South and Central America, and the Caribbean: an archival analysis. Part I. J. Ethnopharmacol. 4, 129–158. 10.1016/0378-8741(81)90032-5 7311595

[B128] Houghton P. J. Manby J. (1985). Medicinal plants of the Mapuche. J. Ethnopharmacol. 13, 89–103. 10.1016/0378-8741(85)90063-7 3990317

[B129] Houghton P. J. Ren Y. Howes M.-J. (2006). Acetylcholinesterase inhibitors from plants and fungi. Nat. Prod. Rep. 23, 181–199. 10.1039/b508966m 16572227

[B130] Hua F. Ha T. Ma J. Li Y. Kelley J. Gao X.. (2007). Protection against myocardial ischemia/reperfusion injury in TLR4-deficient mice is mediated through a phosphoinositide 3-kinase-dependent mechanism. J. Immunol. 178, 7317–7324. 10.4049/jimmunol.178.11.7317 17513782

[B131] Huq M. E. Ikram M. (1968). Alkaloids of *Berberis petiolaris*. Sci. Res. 5, 75–76.

[B132] Hussain K. Shahazad A. Zia-ul-Hussnain S. (2008). An ethnobotanical survey of important wild medicinal plants of Hattar district Haripur, Pakistan. Ethnobot. Leafl. 2008, 5.

[B133] Hussaini F. A. Shoeb A. (1985). Isoquinoline derived alkaloids from *Berberis chitria*. Phytochemistry 24, 633 10.1016/S0031-9422(00)80794-3

[B134] Hutchens A. R. (1992). A Handbook of Native American Herbs: The Pocket Guide to 125 Medicinal Plants and Their Uses. Boston, MA: Shambhala Publications.

[B135] Imanshahidi M. Hosseinzadeh H. (2008). Pharmacological and therapeutic effects of *Berberis vulgaris* and its active constituent, berberine. Phyther. Res. 22, 999–1012. 10.1002/ptr.2399 18618524

[B136] Inbaraj J. J. Kukielczak B. M. Bilski P. Sandvik S. L. Chignell C. F. (2001). Photochemistry and photocytotoxicity of alkaloids from goldenseal (*Hydrastis canadensis* L.) 1. Berberine. Chem. Res. Toxicol. 14, 1529–1534. 10.1021/tx0155247 11712911

[B137] Irvine F. R. (1961). Woody Plants of Ghana. London: Oxford University Press.

[B138] Ishola I. O. Oreagba I. A. Adeneye A. A. Adirije C. Oshikoya K. A. Ogunleye O. O. (2014). Ethnopharmacological survey of herbal treatment of malaria in Lagos, Southwest Nigeria. J. Herb. Med. 4, 224–234. 10.1016/j.hermed.2014.08.001

[B139] Israilov I. A. Yunusov S. (1986). Alkaloids of four species of Argemone. Chem. Nat. Compd. 22, 189–192. 10.1007/BF00598384

[B140] Jayaprakasam R. Ravi T. K. (2014). Development and validation of HPTLC and RP-HPLC methods for the estimation of berberine in *Coscinium fenestratum* extract and its formulation. World J. Pharm. Res. 4, 206–218.

[B141] Jha R. N. Pandey M. B. Singh A. K. Singh S. Singh V. P. (2009). New alkaloids from *Corydalis* species. Nat. Prod. Res. 23, 250–255. 10.1080/14786410801996390 19235025

[B142] Jin C. Shan W. (1982). Quantitative determination of berberine in *Coptis chinensis* by TLC scanner method. Yaoxue Tongbao 17, 145–146.

[B143] Jiofack T. Fokunang C. Guedje N. Kemeuze V. (2009). Ethnobotany and phytomedicine of the upper Nyong valley forest in Cameroon. Afr. J. Pharm. Pharmacol. 3, 144–150.

[B144] Jiofack T. Fokunang C. Kemeuze V. Fongnzossie E. Tsabang N. Nkuinkeu R. (2008). Ethnobotany and phytopharmacopoea of the South-West ethnoecological region of Cameroon. J. Med. Plants Res. 2, 197–206.

[B145] Joshi A. R. Joshi K. (2007). Ethnomedicinal plants used against skin diseases in some villages of Kali Gandaki, Bagmati and Tadi Likhu watersheds of Nepal. Ethnobot. Leafl. 2007, 27.

[B146] Joshi H. R. Kanaki N. (2013). Quantitative analysis of berberine in an ayurvedic formulation-*Rasayana churna* by UV spectrophotometry. J. Pharm. Sci. Biosci. Res. 3, 32–34.

[B147] Jusiak L. (1967). Separation of *Chelidonium majus* alkaloids by countercurrent cascade extraction. II. Acta Pol. Pharm. 24, 65–70.

[B148] Kadiri A. B. (2008). Evaluation of medicinal herbal trade (Paraga) in Lagos State of Nigeria. Ethnobot. Leafl. 2008, 90.

[B149] Kala C. P. (2006). Medicinal plants of the high altitude cold desert in India: diversity, distribution and traditional uses. Int. J. Biodivers. Sci. Manage. 2, 43–56. 10.1080/17451590609618098

[B150] Kamal Y. T. Singh M. Tamboli E. T. Parveen R. Ahmad S. (2011). Quantitative analysis of berberine in *Berberis aristata* fruits and in a traditional anti-inflammatory unani formulation by use of a validated HPLC method. Acta Chromatogr. 23, 157–168. 10.1556/AChrom.21.2013.1.11

[B151] Kamigauchi M. Iwasa K. (1994). *Corydalis* spp.: *in vitro* culture and the biotransformation of protoberberines, in Medicinal and Aromatic Plants VI. Biotechnology in Agriculture and Forestry, Vol 26, ed Bajaj Y. P. S. (Berlin; Heidelberg: Springer), 93–105.

[B152] Karimov A. (1993). *Berberis* alkaloids. Chem. Nat. Compd. 29, 415–438. 10.1007/BF00630564

[B153] Karimov A. Lutfullin K. L. (1986). *Berberis* alkaloids. 2'-*N*-methylisotetrandrine from *Berberis oblonga*. Khimiya Prir. Soedin. 2, 249–251.

[B154] Karimov A. Meliboev S. Olimov V. Shakirov R. (1993). *Berberis alkaloids*. XXX. Dynamics of alkaloid accumulation in *Berberis integerrima* and *B. nummularia*. Khimiya Prir. Soedin. 3, 472–473.

[B155] Karimov A. Shakirov R. (1993). *Berberis* alkaloids. XX. Alkaloids of Berberis iliensis. Khimiya Prir. Soedin. 1, 83–84. 10.1007/BF00631020

[B156] Kariyone T. Koiso R. (1971). Atlas of Medicinal Plants. Osaka: Takeda Chemical Industries.

[B157] Kataoka M. Tokuyama E. Miyanaga Y. Uchida T. (2008). The taste sensory evaluation of medicinal plants and Chinese medicines. Int. J. Pharm. 351, 36–44. 10.1016/j.ijpharm.2007.09.017 17976934

[B158] Kaur C. Miani S. (2001). Fruits and vegetables healthy foods for new millennium. Indian Hort. 45, 29–32.

[B159] Kayode J. (2006). Conservation of indigenous medicinal botanicals in Ekiti State, Nigeria. J. Zhejiang Univ. Sci. B 7, 713–718. 10.1631/jzus.2006.B0713 16909472 PMC1559802

[B160] Khalmatov K. (1964). Khalmatov, Wild-Growing Medicinal Plants of Uzbekistan [in Russian] Tashkent. Meditsina.

[B161] Khamidov I. Faskhutdinov M. Telezhenetskaya M. V. Karimov A. Levkovich M. G. Abdullaev N. D. (1996a). *Berberis* alkaloids. XXXIV. Turcomanine, a new alkaloid from *Berberis turcomanica*. Khimiya Prir. Soedin. 1, 74–76.

[B162] Khamidov I. Karimov A. K. Telezhenetskaya M. V. Tashkhodzhaev B. (1996b). *Berberis* alkaloids. XXXV. *Berberis turcomanica*. Khimiya Prir. Soedin. 1, 107–109.

[B163] Khamidov I. I. Aripova S. F. Karimov A. Yusupov M. M. (1997a). *Berberis* alkaloids. XL. An investigation of the alkaloids of Berberis thunbergii. Chem. Nat. Compd. 33, 599–599. 10.1007/BF02254817

[B164] Khamidov I. I. Aripova S. F. Karimov A. K. (2003). *Berberis* alkaloids. XLI. Alkaloids from leaves of cultivated Berberis oblonga. Chem. Nat. Compd. 39, 407 10.1023/B:CONC.0000003429.41497.b6

[B165] Khamidov I. I. Aripova S. F. Telezhenetskaya M. V. Karimov A. Dzhenberov I. (1997b). *Berberis* alkaloids XXXIX. New alkaloids from B. densiflora. Chem. Nat. Comp. 33, 323–325. 10.1007/BF02234886

[B166] Khamidov I. I. Tashkhodzhaev B. Aripova S. F. Telezhenetskaya M. V. Karimov A. K. (1996c). *Berberis* alkaloids. XXXVII. Study of the alkaloids of *B. oblonga* and *B. integerrima*. Crystal structure of 8-trichloromethyldihydroberberine. Khimiya Prir. Soedin. 6, 889–893.

[B167] Khan I. Najeebullah S. Ali M. Shinwari Z. K. (2016). Phytopharmacological and ethnomedicinal uses of the Genus *Berberis* (Berberidaceae): a review. Trop. J. Pharm. Res. 15, 2047–2057. 10.4314/tjpr.v15i9.33

[B168] Khan M. I. Sri Harsha P. S. C. Giridhar P. Ravishankar G. A. (2011). Berberine and lycopene profiling during the ontogeny of *Tinospora cordifolia* (Willd.) Miers ex Hook. F. & Thoms fruit. Curr. Sci. 100, 1225–1231. 10.3109/09637486.2010.529069 21155657

[B169] Khan S. W. Khatoon S. (2007). Ethnobotanical studies on useful trees and shrubs of Haramosh and Bugrote valleys in Gilgit northern areas of Pakistan. Pak. J. Bot. 39, 699–710.

[B170] Khodzhimatov M. (1989). Dikorastushchiye Lekarstvennuiye Rasteniya Tadzhikistana [Wild-Growing Medicinal Plants of Tadjikistan].

[B171] King J. (1898). King's American Dispensatory. Cincinatti, OH: Ohio Valley Company.

[B172] Kirtikar K. Basu B. (1933). Indian Medicinal Plants, I. Allahabad: Lalit Mohan Basu and Co.

[B173] Kirtikar K. R. Basu B. D. (1998). Indian Medicinal Plants, Vol 1. Allahabad: CSIR publication.

[B174] Kiryakov H. G. Daskalova E. Georgieva A. Kuzmanov B. Evstatieva L. (1982a). Alkaloids from *Corydalis solida* (L.) Swarz. Folia Med. 24, 19–22. 6821055

[B175] Kiryakov H. G. Iskrenova E. Daskalova E. Kuzmanov B. Evstatieva L. (1982b). Alkaloids of *Corydalis slivenensis*. Planta Med. 44, 168–170. 10.1055/s-2007-971432 17402105

[B176] Knapp J. E. Hussein F. T. Beal J. L. Doskotch R. W. Tomimatsu T. (1967). Isolation of two bisbenzylisoquinoline alkaloids from the rhizomes and roots of *Xanthorhiza simplicissima*. J. Pharm. Sci. 56, 139–141. 10.1002/jps.2600560129 6030487

[B177] Končić M. Z. Kremer D. Schühly W. Brantner A. Karlović K. Kalodera Z. (2010). Chemical differentiation of *Berberis croatica* and *B. vulgaris* using HPLC fingerprinting. Croat. Chem. Acta 83, 451–456.

[B178] Kong W. J. Wei J. Zuo Z. Y. Wang Y. M. Song D. Q. You X. F.. (2008). Combination of simvastatin with berberine improves the lipid-lowering efficacy. Metabolism. 57, 1029–1037. 10.1016/j.metabol.2008.01.037 18640378

[B179] Kong Y. Xiao J.-J. Meng S.-C. Dong X.-M. Ge Y.-W. Wang R.-F.. (2010). A new cytotoxic flavonoid from the fruit of *Sinopodophyllum hexandrum*. Fitoterapia 81, 367–370. 10.1016/j.fitote.2009.11.003 19909799

[B180] Kosalec I. Gregurek B. Kremer D. Zovko M. Sanković K. Karlović K. (2009). Croatian barberry (*Berberis croatica* Horvat): a new source of berberine? analysis and antimicrobial activity. World J. Microbiol. Biotechnol. 25, 145–150. 10.1007/s11274-008-9860-x

[B181] Kosina P. Gregorova J. Gruz J. Vacek J. Kolar M. Vogel M.. (2010). Phytochemical and antimicrobial characterization of *Macleaya cordata* herb. Fitoterapia 81, 1006–1012. 10.1016/j.fitote.2010.06.020 20600683

[B182] Kostalova D. Brazdovicova B. Jin H. Y. (1982). Alkaloids from the aboveground parts of Berberis koreana Palib. Farm. Obz. 51, 213–216.

[B183] Kubota M. Katsunori M. Miyazawa Y. (1980). Berberine contents in cultivated *Coptis japonica* Makino. Nagano-ken Eisei Kogai Kenkyusho Kenkyu Hokoku 2, 22–27.

[B184] Kukula-Koch W. Mroczek T. (2015). Application of hydrostatic CCC–TLC–HPLC–ESI-TOF-MS for the bioguided fractionation of anticholinesterase alkaloids from *Argemone mexicana* L. roots. Anal. Bioanal. Chem. 407, 2581–2589. 10.1007/s00216-015-8468-x 25618762 PMC4365284

[B185] Kulkarni S. K. Dhir A. (2010). Berberine: a plant alkaloid with therapeutic potential for central nervous system disorders. Phyther. Res. 24, 317–324. 10.1002/ptr.2968 19998323

[B186] Kunwar R. M. Adhikari N. (2005). Ethnomedicine of Dolpa district, Nepal: the plants, their vernacular names and uses. Lyonia 8, 43–49. 10.1186/1746-4269-2-27

[B187] Küpeli E. Koşar M. Yeşilada E. Hüsnü K. Başer C. (2002). A comparative study on the anti-inflammatory, antinociceptive and antipyretic effects of isoquinoline alkaloids from the roots of Turkish *Berberis* species. Life Sci. 72, 645–657. 10.1016/S0024-3205(02)02200-2 12467905

[B188] Ladino O. J. P. Suárez L. E. C. (2010). Chemical constituents of the wood from *Zanthoxylum quinduense* Tul. (Rutaceae). Quim. Nova 33, 1019–1021. 10.1590/S0100-40422010000500002

[B189] Launert E. (1981). Edible and Medicinal Plants. London: Hamlyn.

[B190] Lee H. Y. Kim C. W. (1999). Isolation and quantitative determination of berberine and coptisine from tubers of *Corydalis ternata*. Saengyak Hakhoechi 30, 332–334.

[B191] Leone M. G. Cometa M. F. Palmery M. Saso L. (1996). HPLC determination of the major alkaloids extracted from *Hydrastis canadensis* L. Phyther. Res. 10, S45–S46.

[B192] Li H. Li X. L. Zhang M. Xu H. Wang C. C. Wang S.. (2014). Berberine ameliorates experimental autoimmune neuritis by suppressing both cellular and humoral immunity. Scand. J. Immunol. 79, 12–19. 10.1111/sji.12123 24354407

[B193] Li W. L. Zheng H. C. Bukuru J. De Kimpe N. (2004). Natural medicines used in the traditional Chinese medical system for therapy of diabetes mellitus. J. Ethnopharmacol. 92, 1–21. 10.1016/j.jep.2003.12.031 15099842

[B194] Li Y.-H. Zhang M. Xiao H.-T. Fu H.-B. Ho A. Lin C.-Y.. (2015). Addition of berberine to 5-aminosalicylic acid for treatment of dextran sulfate sodium-induced chronic colitis in C57BL/6 Mice. PLoS ONE 10:e0144101. 10.1371/journal.pone.0144101 26642326 PMC4671595

[B195] Liu B. Li W. Chang Y. Dong W. Ni L. (2006). Extraction of berberine from rhizome of *Coptis chinensis* Franch using supercritical fluid extraction. J. Pharm. Biomed. Anal. 41, 1056–1060. 10.1016/j.jpba.2006.01.034 16500064

[B196] Liu F. Li Z. Shi X. Zhong M. (2011). Determination of berberine, palmatine and jatrorrhizine in rabbit plasma by liquid chromatography-electrospray ionization-mass spectrometry. J. Pharm. Biomed. Anal. 56, 1006–1015. 10.1016/j.jpba.2011.08.001 21890297

[B197] Liu J. (1992). Extraction of berbamine with water. Zhongguo Yaoxue Zazhi 27, 290–291.

[B198] Liu L. Liu J. Huang Z. Yu X. Zhang X. Dou D.. (2015). Berberine improves endothelial function by inhibiting endoplasmic reticulum stress in the carotid arteries of spontaneously hypertensive rats. Biochem. Biophys. Res. Commun. 458, 796–801. 10.1016/j.bbrc.2015.02.028 25686503

[B199] Liu L. Wang Z. B. Song Y. Yang J. Wu L. J. Yang B. Y.. (2016). Simultaneous determination of eight alkaloids in rat plasma by UHPLC-MS/MS after oral administration of *Coptis deltoidea* C.Y. Cheng et Hsiao and *Coptis chinensis* Franch. Molecules 21, 1–15. 27428938 10.3390/molecules21070913 PMC6274250

[B200] Liu M. Su X. Li G. Zhao G. Zhao L. (2015). Validated UPLC-MS/MS method for simultaneous determination of simvastatin, simvastatin hydroxy acid and berberine in rat plasma: application to the drug-drug pharmacokinetic interaction study of simvastatin combined with berberine after oral administratio. J. Chromatogr. B Anal. Technol. Biomed. Life Sci. 1006, 8–15. 10.1016/j.jchromb.2015.09.033 26519618

[B201] Liu S. Chen Y. Gu L. Li Y. Wang B. Hao J. (2013). Effects of ultrahigh pressure extraction conditions on yields of berberine and palmatine from *Cortex phellodendri amurensis*. Anal. Methods 5, 4506 10.1039/c3ay40784e

[B202] Liu W. Liu P. Tao S. Deng Y. Li X. Lan T.. (2008b). Berberine inhibits aldose reductase and oxidative stress in rat mesangial cells cultured under high glucose. Arch. Biochem. Biophys. 475, 128–134. 10.1016/j.abb.2008.04.022 18471986

[B203] Liu W. H. Hei Z. Q. Nie H. Tang F. T. Huang H. Q. Li X. J.. (2008a). Berberine ameliorates renal injury in streptozotocin-induced diabetic rats by suppression of both oxidative stress and aldose reductase. Chin. Med. J. 121, 706–712. 18701023

[B204] Liu X. Zhang X. Ye L. Yuan H. (2016). Protective mechanisms of berberine against experimental autoimmune myocarditis in a rat model. Biomed. Pharmacother. 79, 222–230. 10.1016/j.biopha.2016.02.015 27044832

[B205] Lou Y. Yuming W. Yanfen D. Jida S. Huang L. (1982). Extractive spectrophotometric determination of berberine. Yaowu Fenxi Zazhi 2, 82–85.

[B206] Lust J. (2014). The Herb Book: The Most Complete Catalog of Herbs Ever Published. New York, NY: Courier Corporation.

[B207] Ma X. Jiang Y. Wu A. Chen X. Pi R. Liu M.. (2010). Berberine attenuates experimental autoimmune encephalomyelitis in C57 BL/6 mice. PLoS ONE 5:e13489. 10.1371/journal.pone.0013489 20976070 PMC2957444

[B208] Maithani A. Parcha V. Kumar D. (2014). Quantitative estimation of berberine content of *Berberis asiatica* from different altitude of Garhwal Himalaya. Asian J. Pharm. Clin. Res. 7, 165–167.

[B209] Majumder B. Schindra S. N. Dutta P. C. (1956). Occurrence of ceryl alcohol in *Argemone mexicana*. J. Indian Chem. Soc. 33, 351–352.

[B210] Manandhar N. P. (2002). Plants and People of Nepal. Portland, OR: Timber Press.

[B211] Manske R. H. F. (1939). The alkaloids of fumariaceous plants. XIX. *Corydalis ophiocarpa* Hook. f. et Thoms. Can. J. Res. Sect. B Chem. Sci. 17, 51–56. 10.1139/cjr39b-009

[B212] Marek R. Seckárová P. Hulová D. Marek J. Dostál J. Sklenár V. (2003). Palmatine and berberine isolation artifacts. J. Nat. Prod. 66, 481–486. 10.1021/np0204996 12713397

[B213] Martinez M. (1984). Las Plantas Medicinales De México, 3rd Edn. Mexico City: CIESAS, Cuadernos de la Casa Chata.

[B214] Martinez O. E. (1977). Flora de Veracruz, Fascí*culo 77* Riverside, CA: University of California.

[B215] Mascareno E. El-Shafei M. Maulik N. Sato M. Guo Y. Das D. K.. (2001). JAK/STAT signaling is associated with cardiac dysfunction during ischemia and reperfusion. Circulation 104, 325–329. 10.1161/01.CIR.104.3.325 11457752

[B216] Meena A. K. Bansal P. Kumar S. (2009). Plants-herbal wealth as a potential source of ayurvedic drugs. *Asian J. Tradit*. Med. 4, 152–170.

[B217] Mell C. D. (1929). Interesting sources of natural dyestuffs. Color 51, 619–820.

[B218] Mikage M. Mouri C. (1999). Pharmacognostical studies of *Berberis* plants (Berberidaceae) from Nepal (1). Altitudinal, interspecific, and partial variations of berberine content in the barks. Sect. Title Pharm. 53, 249–254.

[B219] Mills S. (1985). The Dictionary of Modern Herbalism: A Comprehensive Guide to Practical Herbal Therapy. Wellingborough: Inner Traditions/Bear & Co.

[B220] Minaiyan M. Ghannadi A. Mahzouni P. Jaffari-Shirazi E. (2011). Comparative study of *Berberis vulgaris* fruit extract and berberine chloride effects on acetic acid-induced colitis in rats. Iran. J. Pharm. Res. 10, 97–104. 24363687 PMC3869597

[B221] Misra P. S. Bhakuni D. S. Sharma V. N. Kaul K. N. (1961). Chemical constituents of *Argemone mexicana*. J. Sci. Ind. Res. 20, 186.

[B222] Moerman D. E. (1998). Native American Ethnobotany. Portland, OR: Timber Press.

[B223] Mokgadi J. Turner C. Torto N. (2013). Pressurized hot water extraction of alkaloids in Goldenseal. Am. J. Anal. Chem. 4, 398–403. 10.4236/ajac.2013.48050

[B224] Mølgaard P. Holler J. G. Asar B. Liberna I. Rosenbæk L. B. Jebjerg C. P.. (2011). Antimicrobial evaluation of Huilliche plant medicine used to treat wounds. J. Ethnopharmacol. 138, 219–227. 10.1016/j.jep.2011.09.006 21939748

[B225] Monforte-Gonzalez M. Cecilia G. G. Jorge R. P. Mildred C. P. Vazquez-Flota F. (2012). Berberine and sanguinarine quantitation in *Argemone mexicana* L. (Papaveraceae) tissues by TLC-in situ fluorography. J. Planar Chromatogr. TLC 24, 358–360. 10.1556/JPC.25.2012.4.14

[B226] Montes M. Wilkomirsky T. (1987). Medicina Tradicional Chilena. Concepción: Editiorial de la Universidad de Concepción.

[B227] Muñoz O. (2001). Plantas Medicinales de uso en Chile: Química y Farmacología. Editorial Universitaria.

[B228] Mustafa A. Turner C. (2011). Pressurized liquid extraction as a green approach in food and herbal plants extraction: a review. Anal. Chim. Acta 703, 8–18. 10.1016/j.aca.2011.07.018 21843670

[B229] Musumeci R. Speciale A. Costanzo R. Annino A. Ragusa S. Rapisarda A.. (2003). *Berberis aetnensis* C. Presl. extracts: antimicrobial properties and interaction with ciprofloxacin. Int. J. Antimicrob. Agents 22, 48–53. 10.1016/S0924-8579(03)00085-2 12842327

[B230] Musuyu Muganza D. Fruth B. I. Nzunzu Lami J Mesia G. K. Kambu O. K. Tona G. L.. (2012). *In vitro* antiprotozoal and cytotoxic activity of 33 ethonopharmacologically selected medicinal plants from Democratic Republic of Congo. J. Ethnopharmacol. 141, 301–308. 10.1016/j.jep.2012.02.035 22394563

[B231] Ndenecho E. N. (2009). Herbalism and resources for the development of ethnopharmacology in Mount Cameroon region. Afr. J. Pharm. Pharmacol. 3, 78–86.

[B232] Neuwinger H. D. (1996). African Ethnobotany: Poisons and Drugs: Chemistry, Pharmacology, Toxicology. London: CRC Press.

[B233] Ngono Ngane R. Koanga Mogtomo M. Tchinda Tiabou A. Magnifouet Nana H. Motso Chieffo P. R. Mballa Bounou Z. (2011). Ethnobotanical survey of some Cameroonian plants used for treatment of viral diseases. *Afr. J*. Plant Sci. 5, 15–21.

[B234] Nguimatsia F. Boustie J. Baril F. Amoros M. Girre L. (1998). Les medicaments des pygmees Baka du Cameroun: moeurs therapeutiques, maladies et inventaire des plantes medicinales. Fitoterapia 69, 29–40.

[B235] Noumi E. (2010). Ethno medicines used for treatment of prostatic disease in Foumban, Cameroon. Afr. J. Pharm. Pharmacol. 4, 793–805.

[B236] Noumi E. Anguessin B. (2010). Insecticides and ethnomedicine of HIV/AIDS at Tokombere (Far North Cameroon). Int. J. Pharm. Biomed. Sci. 2, 20–28.

[B237] Noumi E. Yumdinguetmun R. (2010). Plants and treatment of prostatic diseases in Foumban (West Region, Cameroon). Syllab. Rev. 2, 9–16.

[B238] Odugbemi T. O. Akinsulire O. R. Aibinu I. E. Fabeku P. O. (2007). Medicinal plants useful for malaria therapy in Okeigbo, Ondo State, Southwest Nigeria. *Afr. J. Tradit. Complement. Altern*. Med. 4, 191–198. 10.4314/ajtcam.v4i2.31207 PMC2816451 20162091

[B239] Ogbonna D. N. Sokari T. G. Agomuoh A. A. (2008). Antimalarial activities of some selected traditional herbs from Southeastern Nigeria against *Plasmodium* species. Res. J. Parasitol. 3, 25–31. 10.3923/jp.2008.25.31

[B240] Ohemu T. L. Agunu A. Olotu P. N. Ajima U. Dafam D. G. Azila J. J. (2014). Ethnobotanical survey of medical plants used in the traditional treatment of viral infections in Jos, plateau state, Nigeria. *Int. J. Med. Aromat*. Plants 4, 74–81.

[B241] Okunade A. L. Hufford C. D. Richardson M. D. Peterson J. R. Clar A. M. (1994). Antimicrobial Properties of Alkaloids from *Xanthorhiza simplicissima*. J. Pharm. Sci. 83, 404–406. 10.1002/jps.2600830327 8207690

[B242] Oladunmoye M. K. Kehinde F. Y. (2011). Ethnobotanical survey of medicinal plants used in treating viral infections among Yoruba tribe of South Western Nigeria. Afr. J. Microbiol. Res. 5, 2991–3004. 10.5897/AJMR10.004

[B243] Oliver B. E. P. (1960). Medicinal Plants in Nigeria: Being a Course of Four Lectures. Pharmacy Department of the Nigerian College of Arts, Science and Technology, Ibadan.

[B244] Olowokudejo J. D. Kadiri A. B. Travih V. A. (2008). An ethnobotanical survey of herbal markets and medicinal plants in Lagos State of Nigeria. Ethnobot. Leafl. 2008, 116.

[B245] Onwuanibe R. C. (1979). The philosophy of African medical practice. Afr. Issues 9, 25–28. 10.2307/1166259

[B246] Orhan I. Sener B. Choudhary M. I. Khalid A. (2004). Acetylcholinesterase and butyrylcholinesterase inhibitory activity of some Turkish medicinal plants. J. Ethnopharmacol. 91, 57–60. 10.1016/j.jep.2003.11.016 15036468

[B247] Othman M. S. Safwat G. Aboulkhair M. Abdel Moneim A. E. (2014). The potential effect of berberine in mercury-induced hepatorenal toxicity in albino rats. Food Chem. Toxicol. 69, 175–181. 10.1016/j.fct.2014.04.012 24751971

[B248] Pak V. (2005). Medicine plants of folk medicine used for treatment of gastro-intestinal problems in Fergana valley. Korean Food Res. Inst. 18, 150–157.

[B249] Pant N. Garg H. S. Bhakuni K. (1986). Chemical constituents of *B. pseudoumbellata*. Fitoterapia 51, 427–428.

[B250] Park D. W. Jiang S. Liu Y. Siegal G. P. Inoki K. Abraham E.. (2014). GSK3β-Dependent inhibition of AMPK potentiates activation of neutrophils and macrophages and enhances severity of acute lung injury. Am. J. Physiol. 307, L735–L745. 10.1152/ajplung.00165.2014 25239914 PMC4233296

[B251] Park J.-I. Shim J.-K. Do J.-W. Kim S.-Y. Seo E.-K. Kwon H.-J.. (1999). Immune-stimulating properties of polysaccharides from *Phellodendri cortex* (Hwangbek). Glycoconj. J. 16, 247–252. 10.1023/A:1007084506071 10596900

[B252] Parsons H. B. (1882). Examination of the root of *Berberis aquifolium*, v. *alpens*, “oregon grape root.” Pharm. J. 13, 46–48.

[B253] Patel M. C. (2013). Isolation of berberine from *Berberis aristata* by an acid dye method and optimization of parameters. Int. J. Pharm. Sci. Rev. Res. 20, 187–189.

[B254] Pathak N. K. R. Biswas M. Seth K. K. Dwivedi S. P. D. Pandey V. B. (1985). Chemical investigation of *Argemone mexicana*. Pharmazie 40, 202.4023039

[B255] Pěnčíková K. Urbanová J. Musil P. Táborská E. Gregorová J. (2011). Seasonal Variation of Bioactive Alkaloid Contents in *Macleaya microcarpa* (Maxim.) Fedde. Molecules 16, 3391–3401. 10.3390/molecules16043391 21512447 PMC6260594

[B256] Perkin A. G. Hummel J. J. (1895). XLV.—The colouring principle of *Toddalia aculeata* and *Evodia meliaefolia*. J. Chem. Soc. Trans. 67, 413–416. 10.1039/CT8956700413

[B257] Pesman M. W. (1962). Meet Flora Mexicana. Globe, AZ: D.S. King.

[B258] Petcu P. (1965a). Der gehalt an alkaloiden und vitamin C in *Berberis guimpelii*. Planta Med. 13, 178–181. 10.1055/s-0028-1100108

[B259] Petcu P. (1965b). Phytochemical investigation of *Berberis koreana*. Farm. Bucharest, Rom. 13, 21–28.

[B260] Pfoze N. L. Myrboh B. Kumar Y. Rohman R. (2014). Isolation of protoberberine alkaloids from stem bark of *Mahonia manipurensis* Takeda using RP-HPLC. J. Med. Plants Stud. 2, 48–57.

[B261] Phillips R. Foy N. (1990). Herbs. London: Pan Books Ltd.

[B262] Phillips R. Rix M. (1991). Perennials Vol. 1 and 2. London: Pan Books Ltd.

[B263] Phillipson J. D. Gray A. I. Askari A. A. R. Khalil A. A. (1981). Alkaloids From Iraqi Species of Papaveraceae. J. Nat. Prod. 44, 296–307. 10.1021/np50015a011

[B264] Phondani P. C. Maikhuri R. K. Rawat L. S. Farooquee N. A. Kala C. P. Vishvakarma S. C. R. (2010). Ethnobotanical uses of plants among the Bhotiya tribal communities of Niti Valley in Central Himalaya, India. Ethnobot. Res. Appl. 8, 233–244. 10.17348/era.8.0.233-244

[B265] Pilch W. Szygula Z. Tyka A. K. Palka T. Tyka A. Cison T.. (2014). Disturbances in pro-oxidant-antioxidant balance after passive body overheating and after exercise in elevated ambient temperatures in athletes and untrained men. PLoS ONE 9:e85320. 10.1371/journal.pone.0085320 24465535 PMC3896384

[B266] Rahal A. Kumar A. Singh V. Yadav B. Tiwari R. Chakraborty S.. (2014). Oxidative stress, prooxidants, and antioxidants: The interplay. Biomed Res. Int. (2014). 10.1155/2014/761264 24587990 PMC3920909

[B267] Rajan S. Sethuraman M. (1992). *Mahonia leschenaultii*–a toda plant. Anc. Sci. Life 12, 242. 22556593 PMC3336628

[B268] Rajasekaran A. Kumar N. (2009). Rasont – A traditional crude drug prepared from *Berberis* sp and its uses. Indian, J. Tradit. Knowl. 8, 562–563.

[B269] Ransohoff R. M. Hafler D. A. Lucchinetti C. F. (2015). Multiple sclerosis — a quiet revolution. Nat. Rev. Neurol. 11, 134–142. 10.1038/nrneurol.2015.14 25686758 PMC4556342

[B270] Rashid M. H. Malik M. N. (1972). Composition of alkaloids in some *Berberis* species. Pakistan J. For. 22, 43–47.

[B271] Rashmi R. A. Pokhriyal R. Singh Y. (2009). Quantitative Estimation of Berberine in Roots of Different provenances of *Berberis aristata* DC by HPLC and Study of their Antifungal Properties. Pharmacogn. Mag. 5, 355–358. 10.4103/0973-1296.58566

[B272] Richert F. (1918). The extraction of berberine from “michai” (*Berberis darwinii*) and “calafate” (*B. vuxifolia*), in the Argentine. Rev. del Cent. Estud. Agron. y Vet. la Univ. Buenos Aires 11, 11–13.

[B273] Ritch-Krc E. M. Thomas S. Turner N. J. Towers G. H. N. (1996). Carrier herbal medicine: traditional and contemporary plant use. J. Ethnopharmacol. 52, 85–94. 10.1016/0378-8741(96)01392-X 8735452

[B274] Rivera Núñez D. Obon de Castro C. (1996). Ethnopharmacology of Murcia, Actes du 2^*a*^ *Colloque Européen d'Ethnopharmacologei et de la 11*^*a*^ *Conférence internationale d'Ethnomédecine* (Heidelberg), 24.

[B275] Rojsanga P. Gritsanapan W. (2005). Variation of Berberine Content in *Coscinium fenestratum* Stem in Thailand Market. Mahidol Univ. J. Pharm. Sci. 32, 66–70.

[B276] Rojsanga P. Gritsanapan W. Suntornsuk L. (2006). Determination of berberine content in the stem extracts of *Coscinium fenestratum* by TLC densitometry. Med. Princ. Pract. 15, 373–378. 10.1159/000094272 16888396

[B277] Samal P. K. (2013). HPTLC analysis of berberine in *Argemone mexicana*, L. J. Glob. Trends Pharm. Sci. 4, 1073–1076.

[B278] Samhita S. (1963). Sutrasthanam Lakshadi Group. Ed Bhishagratna K. K. Varanasi: Chaukhamba Sanskrit Sansthan.

[B279] San Martín J. (1983). Medicinal plants in central Chile. Econ. Bot. 37, 216–227. 10.1007/BF02858788

[B280] Sandberg F. (1965). Etude sur les plantes medicinales et toxiques d'Afrique equatoriale. 1. Premier inventaire des plantes medicinales et toxiques de la region sudouest de la Republique Centrafricaine et de la region nord de la Republique du Congo/Brazzaville. Cah. la Maboké 3, 5–49.

[B281] Santos A. C. Adkilen P. (1932). The alkaloids of *Argemone mexicana*. J. Am. Chem. Soc. 54, 2923–2924. 10.1021/ja01346a037

[B282] Santra D. K. Saoji A. N. (1971). Phytochemical study of *Argemone mexicana* latex. Curr. Sci. 40, 548–549.

[B283] Saraf G. Mitra A. Kumar D. Mukherjee S. Basu A. (2010). Role of nonconventional remedies in rural India. Int. J. Pharm. Life Sci. 1, 141–159.

[B284] Sasidharan S. Chen Y. Saravanan D. Sundram K. M. Yoga Latha L. (2011). Extraction, isolation and characterization of bioactive compounds from plants' extracts. Afr. J. Tradit. Complement. Altern. Med. 8, 1–10. 22238476 PMC3218439

[B285] Sati S. C. Joshi S. (2011). Aspects of antifungal potential of ethnobotanically known medicinal plants. Res. J. Med. Plants 5, 377–391. 10.3923/rjmp.2011.377.391

[B286] Satija S. Bansal P. Dureja H. Garg M. (2015). Microwave assisted extraction of *Tinospora cordifolia* and optimization through central composite design. J. Biol. Sci. 15, 106–115. 10.3923/jbs.2015.106.115

[B287] Sato F. Yamada Y. (1984). High berberine-producing cultures of *Coptis japonica* cells. Phytochemistry 23, 281–285. 10.1016/S0031-9422(00)80318-0

[B288] Satyavati G. V. Raina M. K. Sharma M. (1987). Medicinal plants of India. New Delhi: Indian Council of Medical Research.

[B289] Schieffer G. W. Pfeiffer K. (2001). Pressurized liquid extraction and multiple, ultrasonically-assisted extraction of hydrastine and berberine from Goldenseal (*Hydrastis canadensis*) with susequent HPLC assay. J. Liq. Chromatogr. Relat. Technol. 24, 2415–2427. 10.1081/JLC-100105948

[B290] Schlotterbeck J. O. (1902). Does *Argemone mexicana* contain morphine? J. Am. Chem. Soc. 24, 238–242. 10.1021/ja02017a006

[B291] Seino Y. Fukushima M. Yabe D. (2010). GIP and GLP-1, the two incretin hormones: similarities and differences. J. Diabetes Investig. 1, 8–23. 10.1111/j.2040-1124.2010.00022.x 24843404 PMC4020673

[B292] Sener B. Temizer H. (1988). Pharmacognosic investigations on *Corydalis solida* (L.) Swartz ssp. *brachyloba* (Boiss.) Cullen & Davis. II. Alkaloids of *Corydalis solida* (L.) Swartz ssp. *brachyloba* (Boiss.) Cullen & Davis. Gazi Univ. Eczac. Fak. Derg. 5, 9–11.

[B293] Sener B. Temizer H. (1990). Chemical Studies on the Alkaloids from *Corydalis solida* subsp. *tauricola*. Planta Med. 56, 510–510. 10.1055/s-2006-961052 17265388

[B294] Sener B. Temizer H. (1991). Chemical studies on the minor isoquinoline alkaloids from *Corydalis solida* subsp. *brachyloba*. J. Chem. Soc. Pakistan 13, 63–66.

[B295] Sezik E. Yesilada E. Shadidoyatov H. Kulivey Z. Nigmatullaev A. M. Aripov H. N.. (2004). Folk medicine in Uzbekistan: I. Toshkent, Djizzax, and Samarqand provinces. J. Ethnopharmacol. 92, 197–207. 10.1016/j.jep.2004.02.016 15138001

[B296] Shah G. M. Khan M. A. (2006). Common medicinal folk recipes of Siran valley, Mansehra, Pakistan. Ethnobot. Leafl. 2006, 5.

[B297] Shahid M. Rahim T. Shahzad A. Latif T. A. Fatma T. Rashid M. (2009). Ethnobotanical studies on *Berberis aristata* DC. root extracts. African, J. Biotechnol. 8, 556–563.

[B298] Sharma P. K. Chauhan N. S. Lal B. (2005). Studies on plant associated indigenous knowledge among Malanis of Kullu district, Himachal Pradesh. Indian J. Trad. Knowl. 4, 403–408.

[B299] Shigwan H. Saklani A. Hamrapurkar P. D. Mane T. Bhatt P. (2013). HPLC method development and validation for quantification of berberine from *Berberis aristata* and *Berberis tinctoria*. Int. J. Appl. Sci. Eng. 11, 203–211.

[B300] Shirwaikar A. Shirwaikar A. Rajendran K. Punitha I. S. R. (2006). *In vitro* antioxidant studies on the benzyl tetra isoquinoline alkaloid berberine. Biol. Pharm. Bull. 29, 1906–1910. 10.1248/bpb.29.1906 16946507

[B301] Singh A. Duggal S. Kaur N. Singh J. (2010). Berberine: Alkaloid with wide spectrum of pharmacological activities. J. Nat. Prod. 3, 64–75.

[B302] Singh A. Lal M. Samant S. S. (2009). Diversity, indigenous uses and conservation prioritization of medicinal plants in Lahaul valley, proposed Cold Desert Biosphere Reserve, India. Int. J. Biodivers. Sci. Manag. 5, 132–154. 10.1080/17451590903230249

[B303] Singh I. P. Mahajan S. (2013). Berberine and its derivatives: a patent review (2009-2012). Expert Opin. Ther. Pat. 23, 215–231. 10.1517/13543776.2013.746314 23231038

[B304] Singh J. Kakkar P. (2009). Antihyperglycemic and antioxidant effect of *Berberis aristata* root extract and its role in regulating carbohydrate metabolism in diabetic rats. J. Ethnopharmacol. 123, 22–26. 10.1016/j.jep.2009.02.038 19429334

[B305] Singh R. Katiyar C. Pasrija A. (2010). Validated HPLC-UV method for the determination of berberine in raw herb Daruharidra (*Berberis aristata* DC), its extract, and in commercially marketed ayurvedic dosage forms. Int. J. Ayurveda Res. 1, 243. 10.4103/0974-7788.76789 21455453 PMC3059448

[B306] Singh R. Tiwari S. S. Srivastava S. Rawat A. K. S. (2012). Botanical and phytochemical studies on roots of *Berberis umbellata* Wall. ex G. Don. Indian J. Nat. Prod. Resour. 3, 55–60.

[B307] Singh S. (2014). Quantitative analysis of Berberine in *Argemone mexicana* Linn. (Papaveraceae) using HPLC and HPTLC. Adv. Plant Sci. 27, 209–211.

[B308] Singh S. S. Pandey S. C. Srivastava S. Gupta V. S. Patro B. Ghosh A. C. (2003). Chemistry and medicinal properties of *Tinospora cordifolia* (Guduchi). Indian J. Pharmacol. 35, 83–91.

[B309] Sir C. C. Chopra I. C. (1958). Indigenous Drugs of India. Kolkata: U.N.Dhar and Sons Private Limted.

[B310] Slavík J. (1978). Characterization of alkaloids from the roots of *Papaver rhoeas* L. Collect. Czechoslov. Chem. Commun. 43, 316–319. 10.1135/cccc19780316

[B311] Slavik J. Slavikova L. (1957). Alkaloide der mohngewächse (Papaveraceae) VIII. Die alkaloide des roten hornmohns (*Glaucium corniculatum* CURT.). *Collect. Czechoslov*. Chem. Commun. 22, 279–285. 10.1135/cccc19570279

[B312] Slavik J. Slavikova L. (1975). Alkaloids of Papaveraceae. LIX. Alkaloids from the leaves of *Bocconia frutescens*. Collect. Czechoslov. Chem. Commun. 40, 3206–3210. 10.1135/cccc19753206

[B313] Slavik J. Slavikova L. Bochorakova J. (1989). Alkaloids of the Papaveraceae. Part LXXXVIII. Alkaloids from *Papaver rhoeas* var. *chelidonioides O. Kuntze, P. confine Jord.*, and *P. dubium L. Collect*. Czechoslov. Chem. Commun. 54, 1118–1125. 10.1135/cccc19891118

[B314] Slavikova L. Slavik J. (1955). Alkaloids of Papaveraceae. VII. *Argemone mexicana*. Chem. List. Pro Vedu a Prum. 49, 1546–1549.

[B315] Slavikova L. Slavik J. (1966). Alkaloide der mohngewächse (Papaveraceae) XXXII. Über die alkaloide aus *Hunnemannia fumariaefolia* SWEET und über die konstitution des alkaloids HF 1. *Collect. Czechoslov*. Chem. Commun. 31, 1355–1362. 10.1135/cccc19661355

[B316] Slavikova L. Tschu S. Slavik J. (1960). Alkaloids of Papaveraceae. XIV. Alkaloids of *Argemone alba. Collect. Czechoslov*. Chem. Commun. 25, 756–760. 10.1135/cccc19600756

[B317] Smyth B. B. (1903). Preliminary list of medicinal and economic kansas plants, with their reputed therapeutic properties. Trans. Kansas Acad. Sci. 18, 191–209. 10.2307/3624794

[B318] Sood P. Modgil R. Sood M. (2010). Physico-chemical and nutritional evaluation of indigenous wild fruit Kasmal, *Berberis lycium* Royle. Indian J. Nat. Prod. Resour. 1, 362–366.

[B319] Srinivasan G. V. Unnikrishnan K. P. Rema Shree A. B. Balachandran I. (2008). HPLC estimation of berberine in *Tinospora cordifolia* and *Tinospora sinensis*. Indian J. Pharm. Sci. 70, 96–99. 10.4103/0250-474X.40341 20390090 PMC2852071

[B320] Srivastava S. K. Rai V. Srivastava M. Rawat A. K. S. Mehrotra S. (2006a). Estimation of heavy metals in different *Berberis* species and its market samples. Environ. Monit. Assess. 116, 315–320. 10.1007/s10661-006-7395-x 16779598

[B321] Srivastava S. K. Rawat A. K. S. Manjoosha S. Mehrotra S. (2006c). Pharmacognostic Evaluation of the Roots of *Berberis chitria* Lindl. Nat. Prod. Sci. 12, 19–23.

[B322] Srivastava S. K. Rawat A. K. S. Srivastava M. (2006b). Pharmacognostic evaluation of the roots of *Berberis chitria*. Nat. Prod. Sci. 12, 19–23.

[B323] Srivastava S. K. Sayyada K. Singh Rawat A. K. Mehrotra S. (2001). Pharmacognostic evaluation of the root of *Berberis aristata* DC. Nat. Prod. Sci. 7, 102–106.

[B324] Srivastava S. K. Singh Rawat A. K. Mehrotra S. (2004). Pharmacognostic evaluation of the root of *Berberis asiatica*. Pharm. Biol. 42, 467–473. 10.1080/13880200490886256

[B325] Srivastava S. K. Rawat A. K. S. (2007). Pharmacognostic evaluation of the roots of *Berberis tinctoria* Lesch. Nat. Prod. Sci. 13, 27–32.

[B326] Steffens P. Nagakura N. Zenk M. H. (1985). Purification and characterization of the berberine bridge enzyme from *Berberis beaniana* cell cultures. Phytochemistry 24, 2577–2583. 10.1016/S0031-9422(00)80672-X

[B327] Stermitz F. (1967). Alkaloids of the Papaveraceae. V. Muramine and berberine from *Argemone squarrosa*. J. Pharm. Sci. 55, 760–762. 10.1002/jps.2600560624 6034814

[B328] Stermitz F. R. Lorenz P. Tawara J. N. Zenewicz L. A. Lewis K. (2000). Synergy in a medicinal plant: antimicrobial action of berberine potentiated by 5'-methoxyhydnocarpin, a multidrug pump inhibitor. Proc. Natl. Acad. Sci. U.S.A. 97, 1433–1437. 10.1073/pnas.030540597 10677479 PMC26451

[B329] Stermitz F. R. Sharifi I. A. (1977). Alkaloids of *Zanthoxylum monophyllum* and *Z. punctatum*. Phytochemistry 16, 2003–2006. 10.1016/0031-9422(77)80113-1

[B330] Stermitz F. R. Stermitz J. R. Zanoni T. A. Gillespie J. (1974). Alkaloids of *Argemone subintegrifolia* and *A. munita*. Phytochemistry 13, 1151–1153. 10.1016/0031-9422(74)80089-0

[B331] Stuart G. A. Smith F. P. (1977). Chinese Materia Medica: Vegetable Kingdom. Shanghai: Gordon Press Publishers.

[B332] Taborska E. Frantisek V. Slavik J. (1980). Alkaloids of the Papaveraceae. LXXI. Alkaloids from *Bocconia frutescens* L. Collect. Czechoslov. Chem. Commun. 45, 1301–1304. 10.1135/cccc19801301

[B333] Tadzhibaev M. M. Zatorskaya I. N. Lutfullin K. L. Shakirov T. T. (1974). Isolation of berberine. Khimiya Prir. Soedin. 10, 48–50. 10.1007/BF00568218

[B334] Tan E. Luo S. Lin S. Tan R. Yu W. Yi Z. (2013). Determination of five active ingredient in *Phellodendron chinensis* var. *glabiusculum* and *P. chinense* by HPLC. Zhongguo Shiyan Fangjixue Zazhi 19, 135–139.

[B335] Tang J. Feng Y. Tsao S. Wang N. Curtain R. Wang Y. (2009). Berberine and Coptidis Rhizoma as novel antineoplastic agents: a review of traditional use and biomedical investigations. J. Ethnopharmacol. 126, 5–17. 10.1016/j.jep.2009.08.009 19686830

[B336] Tang W. Eisenbrand G. (1992). *Corydalis turtschaninovii* Bess. f. yanhusuo YH Chou et CC Hsü, in Chinese Drugs of Plant Origin (Berlin; Heidelberg: Springer), 377–393.

[B337] Tantaquidgeon G. (1928). Mohegan medicinal practices, weather-lore and superstitions. SI-BAE Annu. Rep. 43, 264–270.

[B338] Teng H. Choi O. (2013). Optimum extraction of bioactive alkaloid compounds from *Rhizome coptidis* (*Coptis chinensis* Franch.) using response surface methodology. Solvent Extr. Res. Dev. 20, 91–104. 10.15261/serdj.20.91 24001845

[B339] Thirupurasundari C. J. Padmini R. Devaraj S. N. (2009). Effect of berberine on the antioxidant status, ultrastructural modifications and protein bound carbohydrates in azoxymethane-induced colon cancer in rats. Chem. Biol. Interact. 177, 190–195. 10.1016/j.cbi.2008.09.027 18951886

[B340] Tiwari K. P. Masood M. (1979). Chemical constituents of *Berberis coriaria* Royle. J. Indian Chem. Soc. 56, 310–311.

[B341] Tiwary J. K. Ballabha R. Tiwari P. (2010). Ethnopaediatrics in Garhwal Himalaya. Uttarakhand, India (Psychomedicine Medice). *NY Sci*. J. 3, 123–126.

[B342] Tomè F. Colombo M. L. (1995). Distribution of alkaloids in *Chelidonium majus* and factors affecting their accumulation. Phytochemistry 40, 37–39. 10.1016/0031-9422(95)00055-C

[B343] Tomita M. Kugo T. (1956). Alkaloids of Berberidaceous plants - XIX: Alkaloids of *B. tschonoskyana* I. Isolation of bases. Yakugak Zasshi 79, 317–321. 10.1248/yakushi1947.79.3_317

[B344] Torres R. Villarroel L. Urzua A. Fajardo V. (1992). Constituents of *Berberis congestiflora* and *Berberis horrida*. Fitoterapia 63:376.

[B345] Tsabang N. Fokou P. V. T. Tchokouaha L. R. Y. Noguem B. Bakarnga-Via I. Nguepi M. S. D.. (2012). Ethnopharmacological survey of Annonaceae medicinal plants used to treat malaria in four areas of Cameroon. J. Ethnopharmacol. 139, 171–180. 10.1016/j.jep.2011.10.035 22079831

[B346] Uchiyama T. Kamikawa H. Ogita Z. (1989). Anti-ulcer effect of extract from *Phellodendri cortex*. Yakugaku zasshi J. Pharm. Soc. Japan 109, 672–676. 10.1248/yakushi1947.109.9_672 2607417

[B347] ul Haq I. Hussain M. (1993). Medicinal plants of Mansehra. Hamdard Med. 36, 63–100.

[B348] Uniyal S. K. Singh K. N. Jamwal P. Lal B. (2006). Traditional use of medicinal plants among the tribal communities of Chhota Bhangal, Western Himalaya. J. Ethnobiol. Ethnomed. 2:14. 10.1186/1746-4269-2-14 16545146 PMC1435742

[B349] Uphof J. C. (1959). Dictionary of Economic Plants, 2nd edn. Lehre.

[B350] Uprety Y. Asselin H. Boon E. K. Yadav S. Shrestha K. K. (2010). Indigenous use and bio-efficacy of medicinal plants in the Rasuwa District, Central Nepal. J. Ethnobiol. Ethnomed. 6:3. 10.1186/1746-4269-6-3 20102631 PMC2823594

[B351] Urzúa A. Torres R. Villarroel L. Fajardo V. (1984). Secondary metabolites of *Berberis darwinii*. Rev. Latinoam. Quim. 15, 27–29.

[B352] Usher G. (1974). A Dictionary of Plants Used by Man. London: Constable and Company Ltd.

[B353] Vennerstrom J. L. Klayman D. L. (1988). Protoberberine alkaloids as antimalarials. J. Med. Chem. 31, 1084–1087. 10.1021/jm00401a006 3286870

[B354] Vennerstrom J. L. Lovelace J. K. Waits V. B. Hanson W. L. Klayman D. L. (1990). Berberine derivatives as antileishmanial drugs. Antimicrob. Agents Chemother. 34, 918–921. 10.1128/AAC.34.5.918 2360830 PMC171721

[B355] Versteegh C. P. C. Sosef M. S. M. (2007). Revision of the African genus *Annickia* (Annonaceae). Syst. Geogr. Plants 77, 91–118.

[B356] Vuddanda P. R. Chakraborty S. Singh S. (2010). Berberine: a potential phytochemical with multispectrum therapeutic activities. Expert Opin. Investig. Drugs 19, 1297–1307. 10.1517/13543784.2010.517745 20836620

[B357] Wang C. Li J. Lv X. Zhang M. Song Y. Chen L.. (2009). Ameliorative effect of berberine on endothelial dysfunction in diabetic rats induced by high-fat diet and streptozotocin. Eur. J. Pharmacol. 620, 131–137. 10.1016/j.ejphar.2009.07.027 19686728

[B358] Wang W. Shen Q. Liang H. Hua C. Liu Y. Li F.. (2016). Pharmacokinetic studies of novel berberine derivatives with ultra-performance liquid chromatography–tandem mass spectrometry. J. Chromatogr. B Anal. Technol. Biomed. Life Sci. 1031, 172–180. 10.1016/j.jchromb.2016.07.038 27494281

[B359] Wang Y. Yi X. Ghanam K. Zhang S. Zhao T. Zhu X. (2014). Berberine decreases cholesterol levels in rats through multiple mechanisms, including inhibition of cholesterol absorption. Metabolism 63, 1167–1177. 10.1016/j.metabol.2014.05.013 25002181

[B360] Watt G. (1883). Economic Products of India, Calcutta International Exhibition. Calcuta: Medicinal Products, Superintendent of Government Print.

[B361] Weiner M. A. (1980). Earth Medicine-Earth Food: Plant Remedies, Drugs, and Natural Foods of the North American Indians. New York, NY: Macmillan.

[B362] Willaman J. J. Schubert B. G. (1961). Alkaloid-Bearing Plants and Their Contained Alkaloids (No. 1234). Agricultural Research Service, US Department of Agriculture.

[B363] Wu X. Li Y. Wang Q. Li W. Feng Y. (2015). Effects of berberine and pomegranate seed oil on plasma phospholipid metabolites associated with risks of type 2 diabetes mellitus by U-HPLC/Q-TOF-MS. J. Chromatogr. B Anal. Technol. Biomed. Life Sci. 1007, 110–120. 10.1016/j.jchromb.2015.11.008 26590882

[B364] Xi J. (2015). Ultrahigh pressure extraction of bioactive compounds from plants-a review. Crit. Rev. Food Sci. Nutr. 57, 1097–1106. 10.1080/10408398.2013.874327 25830766

[B365] Xia X. Yan J. Shen Y. Tang K. Yin J. Zhang Y.. (2011). Berberine improves glucose metabolism in diabetic rats by inhibition of hepatic gluconeogenesis. PLoS ONE 6:e16556. 10.1371/journal.pone.0016556 21304897 PMC3033390

[B366] Xiao H. B. Sun Z. L. Zhang H. B. Zhang D. S. (2012). Berberine inhibits dyslipidemia in C57BL/6 mice with lipopolysaccharide induced inflammation. Pharmacol. Rep. 64, 889–895. 10.1016/S1734-1140(12)70883-6 23087140

[B367] Xiao L. Xu N. Guo M. Guo M. Lv B Tao H.. (2014). Berberine protects endothelial progenitor cell from damage of TNF-alpha via the PI3K/AKT/eNOS signaling pathway. Eur. J. Pharmacol. 743, 11–16. 10.1016/j.ejphar.2014.09.024 25257463

[B368] Xu B. Li P. Zhang G. (2015). Comparative pharmacokinetics of puerarin, daidzin, baicalin, glycyrrhizic acid, liquiritin, berberine, palmatine and jateorhizine by liquid chromatography-mass spectrometry after oral administration of Gegenqinlian decoction and active components alignmen. J. Chromatogr. B Anal. Technol. Biomed. Life Sci. 988, 33–44. 10.1016/j.jchromb.2015.01.039 25746576

[B369] Xu K. He G. Qin J. Cheng X. He H. Zhang D.. (2017). High-efficient extraction of principal medicinal components from fresh *Phellodendron* bark (*Cortex phellodendri*). Saudi J. Biol. Sci. 25, 811–815. 10.1016/j.sjbs.2017.10.008 29740248 PMC5936877

[B370] Yang L. Meng X. Yu X. Kuang H. (2017). Simultaneous determination of anemoside B4, phellodendrine, berberine, palmatine, obakunone, esculin, esculetin in rat plasma by UPLC-ESI-MS/MS and its application to a comparative pharmacokinetic study in normal and ulcerative colitis rats. J. Pharm. Biomed. Anal. 134, 43–52. 10.1016/j.jpba.2016.11.021 27875787

[B371] Yang T.-H. (1960a). Alkaloids of Berberidaceae. XXIX. Alkaloids of *Mahonia lomariifolia* and *M. morrisonensis*. Yakugaku Zasshi 80, 1304–1307. 10.1248/yakushi1947.80.9_1304

[B372] Yang T.-H. (1960b). Alkaloids of Berberidaceae. XXVIII. Alkaloids of *Berberis morrisonensis*. Yakugaku Zasshi 80, 1302–1304. 10.1248/yakushi1947.80.9_1302

[B373] Yang T.-H. Lu S.-T. (1960a). Alkaloids of berberidaceous plants. XXV. Alkaloids of *Berberis kawakamii*. 1. Yakugaku Zasshi 80, 847–849. 10.1248/yakushi1947.80.6_847

[B374] Yang T.-H. Lu S.-T. (1960b). Alkaloids of berberidaceous plants. XXVI. Alkaloids of *Berberis mingetsensis*. 1. Yakugaku Zasshi 80, 849–851. 10.1248/yakushi1947.80.6_849

[B375] Yavich P. A. Kakhtelidze M. B. Sarabunovich A. G. (1993). Quantitative determination of berberine in *Phellodendron lavallei* bark. Farmatsiya 42, 49–50.

[B376] Yeung H. (1985). Handbook of Chinese Herbs and Formulas, Vol. 1. Los Angeles, CA: Institute of Chinese Medicine.

[B377] Yin J. Gao Z. Liu D. Liu Z. Ye J. (2008a). Berberine improves glucose metabolism through induction of glycolysis. Am. J. Physiol. Endocrinol. Metab. 294, E148–E156. 10.1152/ajpendo.00211.2007 17971514 PMC2464622

[B378] Yin J. Xing H. Ye J. (2008b). Efficacy of berberine in patients with type 2 diabetes mellitus. Metabolism 57, 712–717. 10.1016/j.metabol.2008.01.013 18442638 PMC2410097

[B379] Yogesh H. S. Chandrashekhar V. M. Katti H. R. Ganapaty S. Raghavendra H. L. Gowda G. K.. (2011). Anti-osteoporotic activity of aqueous-methanol extract of *Berberis aristata* in ovariectomized rats. J. Ethnopharmacol. 134, 334–338. 10.1016/j.jep.2010.12.013 21182919

[B380] Yoo S. J. Lee K. B. Kwak J. H. (1986). Studies on the seasonal variation of berberine contents in *Berberis koreana*. Saengyak Hakhoechi 17, 123–128.

[B381] Yu C. Tan S. Zhou C. Zhu C. Kang X. Liu S.. (2016). Berberine reduces uremia-associated intestinal mucosal barrier damage. Biol. Pharm. Bull. 39, 1787–1792. 10.1248/bpb.b16-00280 27506986

[B382] Zabihullah Q. Rashid A. Akhtar N. (2006). Ethnobotanical survey in kot Manzaray Baba valley Malakand agency, Pakistan. Pak. J. Plant Sci. 12, 115–121.

[B383] Zaha V. G. Qi D. Su K. N. Palmeri M. Lee H. Y. Hu X.. (2016). AMPK is critical for mitochondrial function during reperfusion after myocardial ischemia. J. Mol. Cell. Cardiol. 91, 104–113. 10.1016/j.yjmcc.2015.12.032 26746142 PMC4839186

[B384] Zaman M. B. Khan M. S. (1970). Hundred drug plants of West Pakistan. Medicinal Plant Branch of Pakistan Forest Institute.

[B385] Zeng X. (1999). Relationship between the clinical effects of berberine on severe congestive heart failure and its concentration in plasma studied by HPLC. Biomed. Chromatogr. 13, 442–444. 10.1002/(SICI)1099-0801(199911)13:7<442::AID-BMC908>3.0.CO;2-A 10534753

[B386] Zhang J. Cai C. T. Cai Z. Q. Liu G. Z. Luo Y. Yang Z. X. (2008). Variation patterns of *Coptis teeta* biomass and its major active compounds along an altitude gradient. J. Appl. Ecol. 19, 1455–1461. 18839903

[B387] Zhao X. Zhang J. Tong N. Chen Y. Luo Y. (2012). Protective effects of berberine on Doxorubicin-induced hepatotoxicity in mice. Biol. Pharm. Bull. 35, 796–800. 10.1248/bpb.35.796 22687420

[B388] Zovko Koncić Z. Kremer D. Karlovć K. Kosalec I. (2010). Evaluation of antioxidant activities and phenolic content of *Berberis vulgaris* L. and *Berberis croatica* Horvat. Food Chem. Toxicol. 48, 2176–2180. 10.1016/j.fct.2010.05.025 20488218

